# The loss‐of‐function of *AtNATA2* enhances *At*ADC2‐dependent putrescine biosynthesis and priming, improving growth and salinity tolerance in Arabidopsis

**DOI:** 10.1111/ppl.14603

**Published:** 2024-11-03

**Authors:** Francisco Ignacio Jasso‐Robles, Carlos Eduardo Aucique‐Perez, Sanja Ćavar Zeljković, Iñigo Saiz‐Fernández, Pavel Klimeš, Nuria De Diego

**Affiliations:** ^1^ Czech Advanced Technology and Research Institute (CATRIN), Palacký University Olomouc Olomouc Czech Republic

## Abstract

Putrescine (Put) is a promising small molecule‐based biostimulant to enhance plant growth and resilience, though its mode of action remains unclear. This study investigated the Put priming effect on Arabidopsis mutant lines (*Atadc1, Atadc2, Atnata1*, and *Atnata2*) under control conditions and salinity to understand its role in regulating plant growth.

The *Atadc2* mutant, characterized by reduced endogenous Put levels, showed insensitivity to Put priming without growth enhancement, which was linked to significant imbalances in nitrogen metabolism, including a high Gln/Glu ratio. Contrarily, the *Atnata2* mutant exhibited significant growth improvement and upregulated *AtADC2* expression, particularly under Put priming, highlighting these genes' involvement in regulating plant development.

Put priming enhanced plant growth by inducing the accumulation of specific polyamines (free, acetylated, conjugated, or bound form) and improving light‐harvesting efficiency, particularly in the *Atnata2* line. Our findings suggest that *At*NATA2 may negatively regulate Put synthesis and accumulation via *At*ADC2 in the chloroplast, impacting light harvesting in photosystem II (PSII). Furthermore, the *Atadc2* mutant line exhibited upregulated *AtADC1* but reduced AcPut levels, pointing to a cross‐regulation among these genes. The regulation by *At*NATA2 on *At*ADC2 and *At*ADC2 on *At*ADC1 could be crucial for plant growth and overall stress tolerance by interacting with polyamine catabolism, which shapes the plant metabolic profile under different growth conditions. Understanding the regulatory mechanisms involving crosstalk between *AtADC* and *AtNATA* genes in polyamine metabolism and the connection with certain SMBBs like Put can lead to more effective agricultural practices, improving plant growth, nitrogen uptake, and resilience under challenging conditions.

## 1 INTRODUCTION

Salinity is one of the major challenges for agriculture worldwide, negatively impacting plant morphology, biochemical functions, seed germination, growth, and development, ultimately reducing yields (Arif et al., 2020; Shahzad et al., 2022). Understanding how salinity impacts plant growth and identifying response mechanisms and new technologies to enhance plant tolerance is crucial for vulnerable crops (Bailey‐Serres et al., 2019). Using biostimulants as priming agents to improve plant resilience to stress has recently gained interest in agriculture (Ferrante et al., 2020; Hernandiz et al., 2022b). However, biostimulants are often derived from complex matrices, making it challenging to understand their mechanism of action and potential use as priming agents.

As an alternative, there is a growing interest in studying the function, mode, and mechanism of action of pure substances such as small molecule‐based biostimulants (SMBBs). These substances are particularly interesting because they can be formulated and characterized more quickly than biostimulants derived from by‐products and other biological wastes (De Diego & Spíchal, 2020; Ferrante et al., 2020). This rapid formulation and characterization can accelerate the development of practical solutions for enhancing plant salt stress tolerance.

Several natural plant‐derived compounds can act as priming agents to enhance salinity tolerance in various crop species. These include signaling molecules like hydrogen sulfide (H_2_S), nitric oxide (NO), and compounds such as chitosan, melatonin, ascorbic acid, alpha‐tocopherol, trehalose, phytohormones and polyamines (De Diego & Spíchal, 2020; Murch & Erland, 2021; Niu et al., 2022). The effectiveness of these SMBBs is well‐documented, with studies showing their ability to improve salt stress tolerance during seed pre‐germination metabolism, particularly with polyamines (Colla et al., 2018; De Diego & Spíchal, 2020; Ugena et al., 2018). Polyamines, a group of amine aliphatic compounds, play crucial roles in several stages of plant development and are vital in numerous physiological, cellular, and molecular processes (Chen et al., 2019). The most studied polyamines are putrescine (Put), spermidine (Spd), spermine (Spm), and thermospermine (tSpm). These nitrogen‐rich compounds are abundant in plant tissues and are effective antioxidants, significantly enhancing stress tolerance (Blázquez, 2024). Using polyamines as an SMBB primer agent is a novel and intriguing approach to alleviating the adverse effects of salt stress on crops. However, limited information is available on the mode of action used as a priming treatment. This highlights the need for more in‐depth studies to uncover their mechanism and mode of action.

In the model plant *Arabidopsis thaliana*, Put synthesis occurs through the decarboxylation of the amino acid arginine (Arg), catalyzed by the enzyme arginine decarboxylase (ADC; EC: 4.1.1.19). This is the sole biosynthesis pathway for Put in Arabidopsis, as it lacks the ornithine decarboxylase (ODC; EC: 4.1.1.17) enzyme (Hanfrey et al., 2001). This process produces agmatine (Agm) and *N*‐carbamoyl putrescine as intermediates. Arabidopsis has two distinct *ADC*‐encoding genes, *At*ADC1 and *At*ADC2, each with unique cellular localizations based on their N‐terminal amino acid sequences (Blázquez et al., 2024). These enzymes contain chloroplast‐targeting sequences and endoplasmic reticulum localization, which complicate polyamine biosynthesis regulation (Lou et al., 2020; Maruri‐López & Jiménez‐Bremont, 2017). A double mutant of these genes is lethal (Urano et al., 2005), underscoring the importance of *At*ADC1 and *At*ADC2 in plant development and salt stress response, with *At*ADC2 being particularly crucial (Hummel et al., 2004; Urano et al., 2004, 2005).

Put serves as the precursor for synthesizing higher polyamines: Spd, Spm, and tSpm. These biosynthetic steps involve adding aminopropyl groups provided by *S*‐adenosylmethionine decarboxylases (SAMDC; EC 4.1.1.50). Put acts as the acceptor for the dcSAM‐dependent transfer of aminopropyl groups catalyzed by spermidine synthases (SPDS; EC 2.5.1.16) to produce Spd and then can be used as substrate by spermine synthase (SPMS; EC:2.5.1.22) or thermospermine synthase (ACL5; EC:2.5.1.79) to synthesize Spm and tSpm, respectively. While these higher polyamines are predominantly synthesized in the cytosol, their presence in the nucleus highlights the diverse function of polyamines within the cell (Belda‐Palazón et al., 2012; Bennet et al., 2002; Fuell et al., 2010; Muñiz et al., 2008; Solé‐Gil et al., 2019).

Polyamine metabolism also involves degradation by oxidative enzymes. Copper‐dependent diamine oxidases (DAOs; EC:1.4.3.22) degrade Put and Spd (Planas‐Portell et al., 2013; Tavladoraki et al., 2016), while flavin‐dependent polyamine oxidases (PAOs; EC:1.5.3.11) oxidize Spd, Spm, and tSpm through back‐conversion or terminal catabolic reactions (Angelini et al., 2010; Cona et al., 2006a, 2006b). Back‐conversion reactions oxidize Spd and Spm, producing Put and Spd, respectively, with the simultaneous production of 3‐aminopropanal and hydrogen peroxide (H_2_O_2_). Terminal catabolism reactions use Spd and Spm to generate the aminoaldehydes 4‐aminobutanal or *N*‐(3‐aminopropyl)‐4‐aminobutanal, respectively, also producing 1,3‐diaminopropane (Dap) and H_2_O_2_ (Fincato et al., 2011).

Polyamines can also be acetylated. In mammal cells, this is an essential step in their metabolism, so they must be acetylated to undergo catabolism and transport. Similar acetylation processes are seen in plants (Bewley et al., 2006; Hedge et al., 2007). The enzymes responsible, spermine/spermidine *N*‐acetyl transferases (SSATs) or *N*‐AcetylTransferase Activities (NATAs), belong to the General Control Non‐repressible 5 (GNAT) superfamily of *N*‐acetyltransferases (Bělíček et al., 2023). They transfer an acetyl group from acetyl‐CoA to the amino groups of various substrates, including polyamines (Montemayor & Hoffman, 2008). However, the role of polyamine acetylation in regulating plant salt response and its relationship with synthesis and catabolism remains unclear.

Arabidopsis contains two enzyme isoforms, *N*‐acetyltransferase activity1 (*At*NATA1; EC:2.3.1.‐) and activity2 (*At*NATA2). *At*NATA1 efficiently acetylated thialysine, various polyamines, and ornithine (Orn) and lysine (Lys), producing compounds such as *N*‐acetylputrescine (NAcPut), *N*‐acetylornithine (NAcOrn) and *N*‐acetyllysine (NAcLys). Additionally, *At*ADC1 can convert *N* ^5^‐acetylornithine (N5AcOrn) to NAcPut, indicating evident crosstalk between the activity of *At*ADCs and *At*NATAs in regulating plant development (Lou et al., 2020). Moreover, the accumulation of NAcPut by *At*NATA1 may influence polyamine synthesis and, especially, catabolism, which is crucial for generating H_2_O_2_ and contributing to the defense (Lou et al., 2016). *At*NATA2 prefers thialysine and Dap. This last polyamine can be acetylated or di‐acetylated to N‐acetylDap (NAcDap) or *N, N′*‐ acetylDap (dAcDap), respectively (Jammes et al., 2014). These modifications are biologically relevant, as they antagonize abscisic acid (ABA) to regulate stomata opening (Lou et al., 2020; Lou et al., 2016; Mattioli et al., 2022).

Here, we hypothesized that Put priming‐related stress tolerance involves subtle regulation of polyamine levels through coordinated changes in polyamine synthesis, catabolism, and acetylation. We characterized various Arabidopsis mutant lines involved in Put synthesis and acetylation to test this hypothesis. Combining phenomics, metabolomics, and gene expression analyses, we aimed to understand how Put modulates plant growth under normal and salt stress conditions.

## 2 MATERIALS AND METHODS

### 2.1 Plant material and growth conditions

The experiments were conducted using the model plant *Arabidopsis thaliana* ecotype Columbia (Col‐0) as a wild type (WT) and the following T‐DNA insertional mutant lines: i) Arginine decarboxylase [*adc1‐3* and *adc2‐3* (Cuevas et al., 2008)], and ii) the *N*‐acetyl transferase genes [*Atnata1‐1* (GK‐256F07), *Atnata2‐1* (SALK_092319)]. Standard PCR was performed using specific primers for WT alleles and the T‐DNA sequence to obtain the homozygous lines. For the *Atnata1‐1* mutant line, the primers were LB: 5′‐GAAACAAACTTTAAAGGACCGATC‐3′ and RB: 5′‐GAAACAAACTTTAAAGGACCGATC‐3′ and for the *Atnata2‐1* mutant line, the primers were LB 5′‐TCTAGCTCATATTTTTGAGCATGTG‐3′ and RB 5′‐TACCAAACCCTTTCCTCCTATAAG‐3′. These gene‐specific primers were used in combination with T‐DNA‐specific primers LBb1: 5’‐ATTTTGCCGATTTCGGAAC‐3′ and 8474: 5’‐ATAATAACGCTGCGGACATCTACATTTT‐3′, respectively (Figure S1).

Seeds were surface‐sterilized following the protocol described by De Diego et al. (2017). Then, seeds were sown on square plates containing 0.5× Murashige‐Skoog (MS) (Murashige and Skoog 1962) medium (Duchefa Biochemie) at pH 5.7, supplemented with 0.6% Phytagel (Sigma–Aldrich) as a gelling agent. Half of the seeds were germinated with 0.1 mM Put (Sigma‐Aldrich) added as a priming agent to the germination culture medium, and the remaining seeds were germinated without supplementation. The plates were kept for three days at 4°C in the dark, then transferred into a growth chamber (PERCIVAL) under controlled conditions: 22°C, 16/8 h light/dark cycle, and a photon irradiance of 120 μmol photons of PAR m^−2^ s^−1^) and placed vertically.

### 2.2 Phenotyping analysis

Three‐day‐old seedlings of similar size were transferred under sterile conditions into 48‐well plates containing full MS media. One seedling per well was placed in the plates and immediately sealed with perforated transparent foil to allow gas exchange.

Two independent experiments were conducted to understand the mode of action induced by Put priming in Arabidopsis under optimal and salt stress conditions: (1) WT and four mutant lines were grown in full MS, with half of them coming from seeds primed with 0.1 mM Put, and (2) selected lines grown in full MS alone (optimal conditions) or supplemented with 100 mM NaCl (PENTA) to induce salt stress, with half of them also coming from seeds primed with 0.1 mM Put. Each growth condition included an equal number of seedlings with and without Put priming, distributed in two 48‐well plates (one seedling per well), resulting in 96 seedlings as biological replicates. All 48‐well plates were transferred to the PlantScreen™ XYZ system (Photon Systems Instruments). The conditions were set to simulate a long‐day regime with 22/20°C in a 16/8 h light/dark photoperiod cycle, an irradiance of 120 μmol photons of PAR m^−2^ s^−1^, and a relative humidity of 60%, as described by De Diego et al. (2017).

Plant growth dynamics were monitored using a top‐view RGB camera that captured images twice daily (at 9 a.m. and 3 p.m.) for 8 days. After image processing, morphological parameters such as rosette area and perimeter (measured in green pixels) per day were determined for each Arabidopsis line under different priming and growth conditions (Ugena et al., 2018).

On the last day of the experiment, top‐view FluorCam images were automatically taken from the 48‐well plates in the PlantScreen™ Compact system at the Olophen platform. The FluorCam imaging system has an LED light panel and a high‐speed charge‐coupled device camera (720 × 560‐pixel resolution, 50 fps, and 12‐bit depth). Modulated light of a known wavelength was used to detect the ChlF signal. Three types of sources were used: (1) PAM short‐duration measuring flashes (33 μs) at 618 nm, (2) orange‐red (618 nm) and cool‐white (6,500 K) actinic lights with maximum irradiance of 440 μmol m^−2^ s^−1^ and (3) saturating cool‐white light with maximum irradiance of 3,000 μmol m^−2^ s^−1^ (Sorrentino et al., 2021). The well plates were automatically loaded into the system light‐isolated imaging cabinet with a top‐mounted LED light panel. After the 45‐minute dark adaptation period, when PSII reaction centers opened, the well plates were automatically transported to the ChlF imaging cabinet. A 5 s flash of light at 0.5 μmol m^−2^ s^−1^ intensity was applied to measure the minimum fluorescence (F_0_), followed by a saturation pulse of 0.8 s at 6,000 μmol m^−2^ s^−1^ to determine the maximum fluorescence (F_m_).

Plants were relaxed in the dark for 3 s and then subjected to 70 s cool‐white actinic light to drive photosynthesis and measure the peak rise in fluorescence (F_p_). Additional saturation pulses were applied at 8, 18, 28, 48, and 68 s during actinic illumination for 3 min, corresponding to L_1_, L_2_, L_3_, L_4_ and Lss states at 500 μmol m^−2^ s ^−1^ constant photon irradiance to obtain the light‐adapted initial fluorescence (F_0_’) and steady state‐fluorescence yield (F_t_). A final saturating blue light pulse (6,000 μmol m^−2^ s^−1^, 0.8 s) was applied to measure the maximum fluorescence in the light‐adapted state (F_m_′), and the level of ChlF was measured just before the saturation pulse was considered the steady‐state fluorescence in the light‐adapted state (F_t_).

The PlantScreen™ Data Analyser calculated several parameters: maximum photosystem II (PSII) quantum yield (QY_max_ = F_v_/F_m_), steady‐state PSII quantum yield (QY = F_m_′‐F_t_/F_m_′), PSII quantum yield of the light‐adapted sample at steady‐state (F_v_/F_m_ = F_m_′‐F_0_’/F_m_′), steady‐state non‐photochemical quenching (NPQ = F_m_‐F_m_′/F_m_′), coefficient of photochemical quenching in the steady‐state estimate of the fraction of open PSII reaction centers PSII_open_/(PSII_open_ + PSII_closed_) [*q*_P_ = ((F_m_′‐F_t_)/(F_m_′‐F_0_’)], coefficient of non‐photochemical quenching in steady‐state (*q*_N_ = F_m_‐F_m_′/F_m_), the fraction of ‘open' reaction centers [*q*_L_ = (F_m_’‐F_t_)/(F_m_’‐F_0_’) × (F_0_’/F_t_)], and electron transference rate (ETR = QY × PAR × α × *f*) where *f* (0.5) accounts for the energy partitioning between PSII and PSI, and indicates that the excitation energy is distributed equally between the two photosystems; and α (0.84) is the leaf absorbance by the photosynthetic tissues (Maxwell & Johnson, 2000).

After the phenotyping study, the samples were collected, immediately frozen in liquid nitrogen, and stored under −80°C until the analysis of metabolomic profiles and gene expression.

### 2.3 Seed viability bioassay

Seed germination bioassay followed the protocol described by Pouvreau et al. (2013), with modifications by Ugena et al. (2018). Arabidopsis seeds were sterilized using 70% ethanol with 0.01% Triton X‐100 solution. After thorough washing with sterile water, 10 g/L of seeds from each Arabidopsis line were stratified in 1 mM HEPES solution (pH 7.5), with or without 100 μM Put as a priming agent, and kept at 4°C in the dark for four days. Following stratification, half of the solution was discarded, and 0.1% agarose and 1 mM HEPES were added to keep a final seed concentration of 10 g/L. The agarose prevents seed sedimentation and allows for pipetting homogeneous seed concentration per well. A total of 50 μL of the solution containing 20–40 seeds per well was pipetted into 96‐well plates, followed by 30 μL of sterile water and 20 μL of 500 mM NaCl (or sterile water for controls) added to each well. Eight plates were used per genotype and treatment.

Plates were sealed with gas‐permeable foil and incubated under controlled conditions with a 16/8 h light/dark photoperiod at 22°C and light intensity of 110 μmol m^−2^ s^−1^ for 48 h. After this period, 10 μL of 5 g/L methyl thiazolyl diphenyl‐tetrazolium bromide (MTT) was added to each well. The plates were resealed and returned to the growth chamber for 24 h. Afterward, the plates were scanned to determine the seed viability, and the percentage of stained seeds was calculated. Eight biological replicates were used: eight wells per variant, one well plate per line.

### 2.4 Target metabolic analysis

The endogenous levels of free amino acids, free, conjugated, and bound polyamines, and acetylated polyamines and amino acids were determined in lyophilized plant material of Arabidopsis rosettes following the protocol described by Ćavar Zeljković et al. (2024). Briefly, the 96 Arabidopsis seedlings used as biological replicates for the phenotypic traits were randomly collected into four independent groups. Each group was pooled separately as biological replicates (*n* = 4) for metabolic and gene expression analysis and lyophilized before analysis. 3–5 mg of lyophilized plant material was extracted with 1 mL of 50% EtOH. Three aliquots were used to quantify the different metabolic groups: (1) 200 μL for free amino acids and acetylated compounds, (2) 250 μL for free polyamines, and (3) an additional 250 μL for conjugated polyamines. The remaining pellet was used to extract the bound polyamine fractions. The aliquot (3) was subjected to acid hydrolysis with 100 μL of 12 M HCl for 16 h at 40°C to liberate the conjugated polyamines. The pellet was hydrolyzed by treatment with 200 μL of 6 M NaOH under sonication for 15 min, followed by heating at 40°C in 200 μL of 12 M HCl to release macromolecule‐bound polyamines. The three independent fractions were then derivatized by benzoylation. All samples were analyzed in a Nexera x2 UHPLC instrument (Shimadzu Handels GmbH) coupled to an MS‐8050 mass spectrometer. Chromatographic separation was performed using Acquity UPLC BEH C18 (50 × 2.1 mm; 1.7 μm particle size) analytical column with a suitable pre‐column. The column was maintained at 40°C, and the flow rate was 0.4 mL min^−1^. The injection volume was 2 μL.

### 2.5 Gene expression

The expression of the genes involved in polyamine biosynthesis, catabolism, and acetylation was determined in the same four independent pools of Arabidopsis seedlings used as biological replicates for the targeted metabolomics. Total RNA was isolated from lyophilized Arabidopsis rosettes using TRI reagent (Sigma‐Aldrich) and treated with the Turbo DNase‐free kit (Ambion) to eliminate genomic DNA. cDNA synthesis was performed using the gb Elite Reverse Transcription Kit according to the manufacturer's instructions (Generi Biotech). Diluted cDNA samples were used as qPCR templates with gb SG PCR Master Mix (Generi Biotech) and 300 nM specific primers on a QuantStudio™ 7 Pro Real‐Time PCR System. The details of the oligonucleotides used are provided in Table S1. Gene expression was normalized using *AtUBQ10* as the reference gene. The fold change in gene expression relative to control samples was calculated using the 2^−ΔΔCt^ method (Livak & Schmittgen, 2001).

### 2.6 Data Analysis

Phenotyping, metabolic profile, and gene expression data from experiments were analyzed in a completely randomized design. Fluorescence parameters and metabolic profile data were log_2_‐transformed to normalize regarding the WT line under control and stress conditions. Metabolic and gene expression data were analyzed using two‐way ANOVAs to evaluate the interaction between line and growth conditions, followed by Tukey's test (*p* < 0.05) to understand better the differences between the mutant lines and WT.

Principal component analysis (PCA) on log_2_‐transformed data was used to determine the relationship between the parameters, metabolites, and genes with the evaluated factors. Data analysis was performed using RStudio software (RStudio 2023.12.1 + 402 “Ocean Storm” Release, 2024‐01‐28), with the packages *factoextra, agricolae, ggplot2,* and *corrplot*.

## 3 RESULTS

### 3.1 Put priming enhanced biomass production in all Arabidopsis lines except the *Atadc2* mutant under optimal growth conditions

**FIGURE 1:**
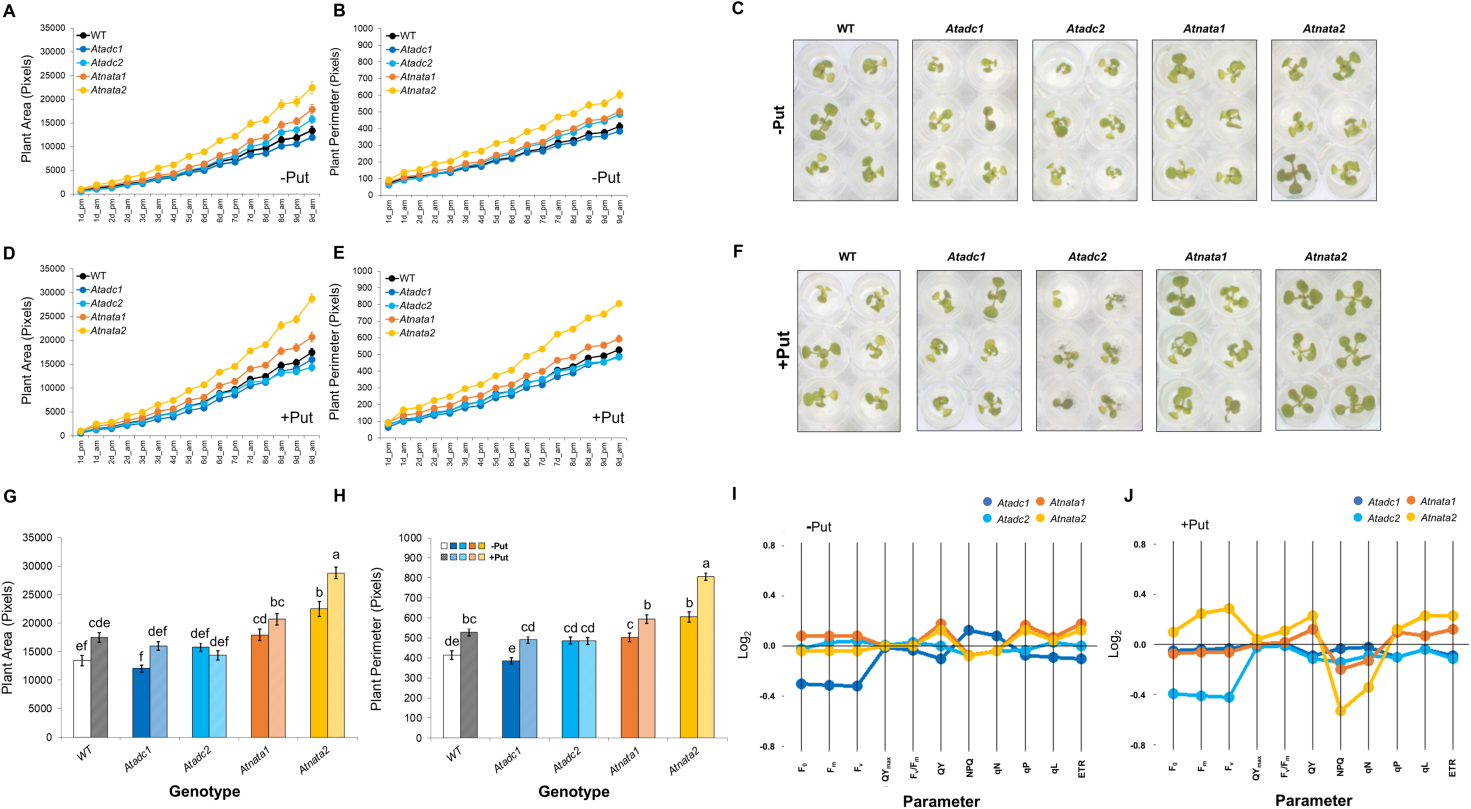
**Effect of Put priming on the biomass production and chlorophyll fluorescence‐related parameters in the WT, *Atadc*, and *Atnata* mutant lines.** Phenotyping analysis of Arabidopsis mutant lines *Atadc1*, *Atadc2*, *Atnata1*, and *Atnata2* and WT under control (full MS) after priming with (+) or without (−) 0.1 mM Put for three days. RGB images were taken twice daily for 7 days. Arabidopsis rosette area and perimeter (green pixels), and RGB images of the last day of the experiment for all lines without **(A‐C)** or with **(D‐F)** Put priming. Final rosette area **(G)** and perimeter **(H)** (green pixels) of the five Arabidopsis lines on the last day of measurement. Chlorophyll fluorescence‐related parameters of the five Arabidopsis lines without or with Put priming on the last day of experiment **(I‐J)**. The values represent the average of the 96 biological replicates per variant (line x priming). Statistical analysis was performed using two‐way ANOVA (*p* < 0.05) followed by a Tukey's test.

Dynamic changes in the Arabidopsis rosette area and perimeter were extracted from the RGB images. *Atnata1* and, notably, *Atnata2* mutant lines exhibited a larger rosette area and perimeter than WT, as indicated by a better‐growing curve (Figure 1A‐C; Table S2). Put priming generally enhanced the rosette area and perimeter in all lines except for *Atadc2*, particularly for the perimeter (Figure 1D‐F). By the end of the experiment, significant differences in biomass among the lines and due to the priming effect were observed (Figure 1G, H; Table S2). The effect was particularly evident in the two *Atnata* lines, with rosette area increases of 33 and 67% and perimeter increases of 21% and 46% for the *Atnata1* and *Atnata2* mutants, respectively, compared to WT.

Interestingly, the *Atadc1* and *Atadc2* mutant lines had lower seed viability than WT under all tested growth conditions. However, priming with Put restored seed viability to WT levels (Figure S2A). These results suggest distinct roles and responses to Put priming of the *AtADC* genes regulating germination and seedling growth in Arabidopsis.

### 3.2 Seed priming with Put reduced photosynthetic capacity in the *Atadc2* line but enhanced in *Atnata2* under optimal conditions

No differences in fluorescence‐related parameters were observed among the lines under optimal conditions (Figure 1H, Supplementary File S1A). Only the *Atadc1* line reduced F_0_, F_m_, F_v_, F_v_/F_m_, QY, *q*_P_, *q*_L_, and ETR along with increased non‐photochemical dissipation (NPQ and *q*_N_) compared to WT (Figure 1H, Supplementary File S1A). Seed priming with Put removed the differences between these two lines (Figure 1I). However, Put priming negatively affected the fluorescence‐related parameters in *Atadc2*, reducing F_m_, F_v_, QY_max_, F_v_/F_m_, QY, *q*_P_, *q*_L_, and ETR parameters, and increasing NPQ and *q*_N_ compared to WT. Conversely, the *Atnata2* mutant line improved the fluorescence‐related parameter after Put priming (Figure 1I, Supplementary File S1A). Overall, Put priming significantly enhanced biomass and physiological traits in *Atnata2*, highlighting this gene as a negative mediator of plant growth and photosynthesis capacity in Arabidopsis. In contrast, Put priming negatively impacted the *Atadc2* line at the seedling stage but significantly improved seed viability, suggesting that the *AtADC2* gene is necessary for mediating Put priming‐induced growth at seedling stages but not for germination.

### 3.3 Put priming impact on the N‐related metabolites

**FIGURE 2:**
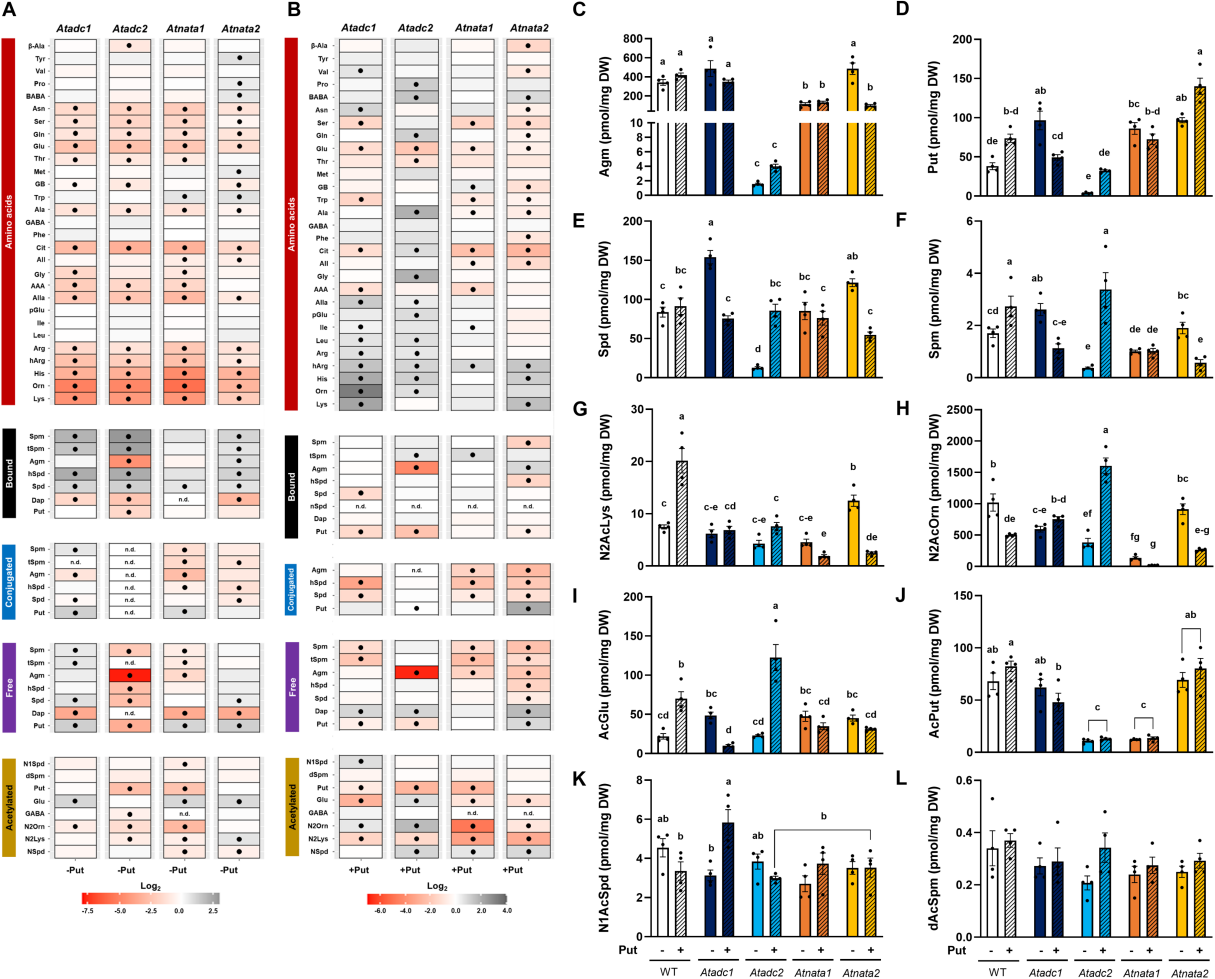
**Metabolic changes induced by Put priming in the WT, *Atadc,* and *Atnata* mutant lines.** Metabolite content related to N‐containing metabolites was analyzed in the rosettes of five Arabidopsis lines: WT (Col0) and four mutant lines (*Atadc1*, *Atadc2*, *Atnata1*, and *Atnata2*) with or without priming with 0.1 mM Put. Heatmap **(A)** representing the changes of free amino acids [β‐alanine (β‐Ala), tyrosine (Tyr), valine (Val), proline (Pro), β‐aminobutyric acid (BABA), asparagine (Asn), serine (Ser), glutamine (Gln), glutamic acid (Glu), threonine (Thr), methionine (Met), betaine (Bet), tryptophan (Trp), alanine (Ala), γ‐aminobutyric acid (GABA), phenylalanine (Phe), citrulline (Cit), glycine (Gly), aspartic acid (AAA), D‐aspartic acid (DAA), pyroglutamic acid (pGlu), isoleucine (Ile), leucine (Leu), arginine (Arg), homo‐arginine (hArg), histidine (His), ornithine (Orn), lysine (Lys)] and bound, conjugated, free and acetylated polyamines [putrescine (Put), spermidine (Spd), spermine (Spm), 1,3‐diaminopropane (DAP), homospermidine (hSpd)] in the mutant lines compared to WT without **(A)** or with **(B)** Put priming. The asterisk (*p* < 0.05*) indicates significant differences between the mutant and WT lines. Changes in the content of Agm **(C)**, Put (**D),** Spd (**E)** Spm **(** **F)** N2AcLys (**G)**, N2AcOrn **(H)**, AcGlu **(** **I)**, AcPut **(J)**, N1AcSpd **(K)** and dAcSpm **(L)** levels in the five Arabidopsis lines with or without Put priming. Different letters indicate significant differences according to Tukey's test after two‐way ANOVA (*p* < 0.05). Mean ± SE; *n* = 4 (each biological replicate consisted of an independent pool of 24 seedlings).

To understand further the mechanism of action induced by Put priming in Arabidopsis seedlings, a targeted metabolomic analysis was conducted to quantify N‐related compounds. This analysis was performed on the collected seedlings at the end of the experiment. Without priming, all mutant lines generally had lower free amino acid content than the WT, especially those related to N metabolisms such as glutamate (Glu), glutamine (Gln), asparagine (Asn), or alanine (Ala), and many precursors of polyamine biosynthesis (Figure 2A, Supplementary File S2A)— specific metabolites accumulated only in the *Atnata* lines. For instance, both mutant lines had higher tryptophane (Trp) levels than WT, with the *Atnata2* mutant line also showing higher content of tyrosine (Tyr), β‐aminobutyric acid (BABA), Pro, and Met (Figure 2A, Supplementary File S2A). Notably, the significant reduction of Gln and Glu in the four mutant lines ended with higher Gln/Glu ratios, particularly in *Atadc1* and *Atadc2,* with values of 27.9 and 24.3 compared to the WT values of 10.7 (Supplementary Figure S3 and File S2A). This imbalance in the N fixation could also affect their morphology and physiology, impacting other metabolic pathways.

Put priming induced the opposite response, enhancing the content of specific amino acids in the mutant lines compared to WT (Figure 2B, Supplementary File S2A). The increase in free amino acid content was more pronounced in the *Atadc* mutant lines and, to a lesser extent, in *Atnata* ones. Specifically, amino acid precursors for polyamine synthesis, such as Arg, Orn, hArg, and His, accumulated in Put‐primed *Atadc1* and *Atnata2* mutants (Figure 2B). Additionally, the primed *Atatdc2* mutant line also increased the Met and Cit levels, amino acids involved in polyamine metabolism, and Ala, BABA, and proline (Pro), among other N‐related compounds like allantoic acid (Alla) and pyroglutamate (pGlu) (Figure 2B, Supplementary File S2). Furthermore, the Gln levels in the Put‐primed *Atadc2* line increased the already high Glu/Gln ratio to 76.6 (Figure 2B, Supplementary File S2A), indicating a substantial Put‐priming induced alteration on its N metabolism. This demonstrates a clear impact of the *AtADC2* gene and its connection to Put on the N metabolism of Arabidopsis seedlings.

### 3.4 *Atnata* and *Atadc* mutant lines varied in the polyamine pool, which was modified by Put priming

To further understand the involvement of the Arabidopsis *AtADC* and *AtNATA* genes in plant growth, we quantified the content of bound, conjugated, and free polyamines in seedlings with and without Put priming. Under no priming conditions, bound Spm, tSpm, hSpd, and Spd levels significantly increased in the *Atadc1*, *Atadc2*, and *Atnata2* mutant lines compared to WT, while bound Dap was reduced (Figure 2A, Supplementary File S2A). The *Atnata1* line did not change the bound polyamines compared to WT, except for a significant increase in bound Spd (Figure 2A). The *Atadc2* line showed the most significant reduction in bound Agm and Put under no priming conditions (Figure 2A, Supplementary File S2A). Interestingly, Put priming reduced the difference in bound polyamines between the mutant lines and WT (Figure 2B). Only the *Atadc2* and *Atnata1* lines had significantly higher bound tSpm levels, and the *Atnata2* mutant enhanced the bound Agm compared to Put‐primed WT (Figure 2B, Supplementary File S2A).

Conjugated polyamines were under the detection level in the *Atadc2* mutant line without priming (Figure 2A, Supplementary File S2A). They were also reduced in the two *Atnata* mutant lines and, to a lesser extent, in the *Atadc1* line, compared to WT. However, the *Atadc1* mutant line accumulated more conjugated Spm and Put. Priming did not alter the profile of the mutant lines, which maintained reduced levels of conjugated polyamines compared to WT (Figure 2B). The most notable result was the significant accumulation of conjugated Put in *Atadc2* and *Atnata2* compared to WT as an effect of Put priming.

The content of free polyamines also varied among lines and as an effect of Put priming (Figure 2A‐C). Two different profiles were observed without priming: *Atadc1* and *Atnata2*, which tended to accumulate the free polyamine forms, and *Atnata1*, and particularly, *Atadc2,* which reduced them (Figure 2, Supplementary File S2A). For instance, the *Atadc2* and *Atnata1* lines showed reduced free Agm, tSpm, and Spm levels compared to WT (Figure 2A, C, and F). They also showed a lower accumulation of free Dap than WT (Figure 2A, Supplementary File S2A). However, the seed priming with Put enhanced the content of free Dap in all mutant lines compared to WT except for *Atnata1* (Figure 2B). The most significant priming effect was observed in *Atnata2*, reducing the rest of the free detected polyamines except Put (Figures 2B‐F, Supplementary File S2A). Overall, it was clear that all mutant lines had affected the polyamine metabolism differently. However, they presented a common response: the reduction of Dap, a final product of polyamine catabolism. This response was modified by Put priming, indicating a clear cross‐regulation between certain compounds related to polyamine synthesis and catabolism.

### 3.5 *Atadc* and *Atnata* mutant lines differed in the content of acetylated compounds compared to WT, particularly after Put priming

It is well‐known that *At*NATAs acetylate free polyamines and amino acids in plants (Bělíček et al., 2023; Lou et al., 2016; Mattioli et al., 2022). We analyzed several acetylated forms to understand better the roles of the two *At*NATA isoforms in Arabidopsis and how they regulate plant growth (Figure 2). Similar tendencies were observed for concrete acetylated compounds in the mutant lines compared to WT in the seedling without priming (Figure 2A and B, Supplementary File S2A). For instance, all mutant lines had higher AcGlu levels than WT, except for *Atadc2,* which did not show significant differences (Figure 2A). However, the N2AcOrn levels were reduced, especially in the *Atnata1* line, but not *Atnata2*.

Seed priming with Put modified the content of acetylated compounds and altered the differences with WT. The *Atadc* lines, particularly *Atadc2,* accumulated more N2AcOrn, whereas *Atnata* lines showed significantly reduced content (Figure 2G, Supplementary File S2A). Put priming also tripled the N2AcLys content in WT, lowering it in the *Atnata* mutant lines, especially in *Atnata2*. The acetylated form of the superior polyamines, Spd and Spm, did not vary under any conditions (Figure 2I, J). Only N1AcSpd accumulated in the *Atadc1* plants when the seeds were primed with Put compared to WT at the same conditions (Figure 2I, Supplementary File S2A).

### 3.6 *Atadc2* and *Atnata2* mutant lines differed at phenotypic and metabolic levels and responded oppositely to Put priming

**FIGURE 3:**
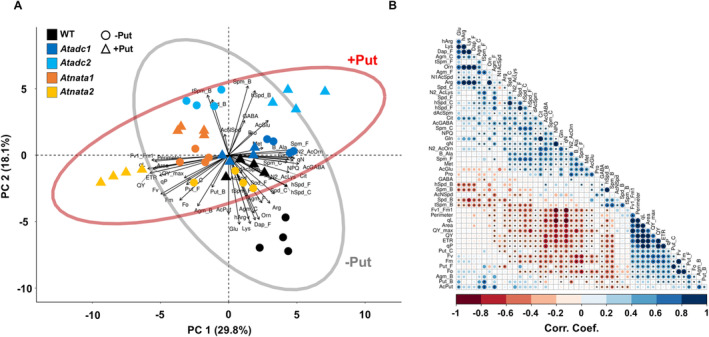
**Multivariate statistical analysis of metabolites, phenotyping, and chlorophyll fluorescence parameters in the *Atadc* and *Atnata* mutant lines primed with Put.** Principal component (PC) analysis **(A)** and correlation matrix **(B)** of the N‐containing metabolites, rosette area and perimeter, and chlorophyll fluorescence‐related parameters in WT and four Arabidopsis mutant lines (*Atadc1*, *Atadc2*, *Atnata1*, and *Atnata2*) without or with 0.1 mM Put priming. The percentages of total variance represented by PC1 and PC2 are shown in parentheses.

To better visualize and understand the differences among lines, we performed multivariate statistical analysis, including principal component (PC) analysis and a correlation matrix (Figure 3). The first two PC1 and PC2 explained 47.9% of the total variance and primarily separated non‐primed WT from the *Atadc2* mutant line (with or without treatment) and Put‐primed *Atnata2* (Figure 3A). Put‐primed *Atnata2* exhibited a higher rosette area and perimeter, traits that were positively correlated with fluorescence‐related parameters such as QY_max_, QY, F_v_’/F_m_′, and ETR, and metabolites like Put in its four forms (free, conjugated, bound, and acetylated) (Figure 3). Conversely, this line presented the lowest values of non‐photochemical quenching‐related parameters (NPQ and *q*_N_), which were positively related to the accumulation of many metabolites involved in polyamine biosynthesis and higher polyamines such as Spd, hSpd, and Spm in their free or conjugated forms, along with certain acetylated compounds like AcGABA, N2AcOrn, N2AcLys and dAcSpm (Figure 3). Interestingly, WT and the *Atadc2* mutant line were on opposite sides of the PCA plot (Figure 3A). Non‐primed WT had higher levels of Glu, Lys, hArg, Orn, free and conjugated Agm, and free tSpm and Dap, whereas *Atadc2* exhibited the highest pool of bound polyamines and γ‐aminobutyric acid (GABA), independent of the priming. These results highlighted the *Atnata2* and *Atadc2* mutant lines as having the most significant phenotypic and metabolic differences compared to WT, with an opposite response to Put priming.

### 3.7 Seed priming with Put enhanced biomass production in the *Atnata2* mutant lines but not in *Atadc2* under salt stress

**FIGURE 4:**
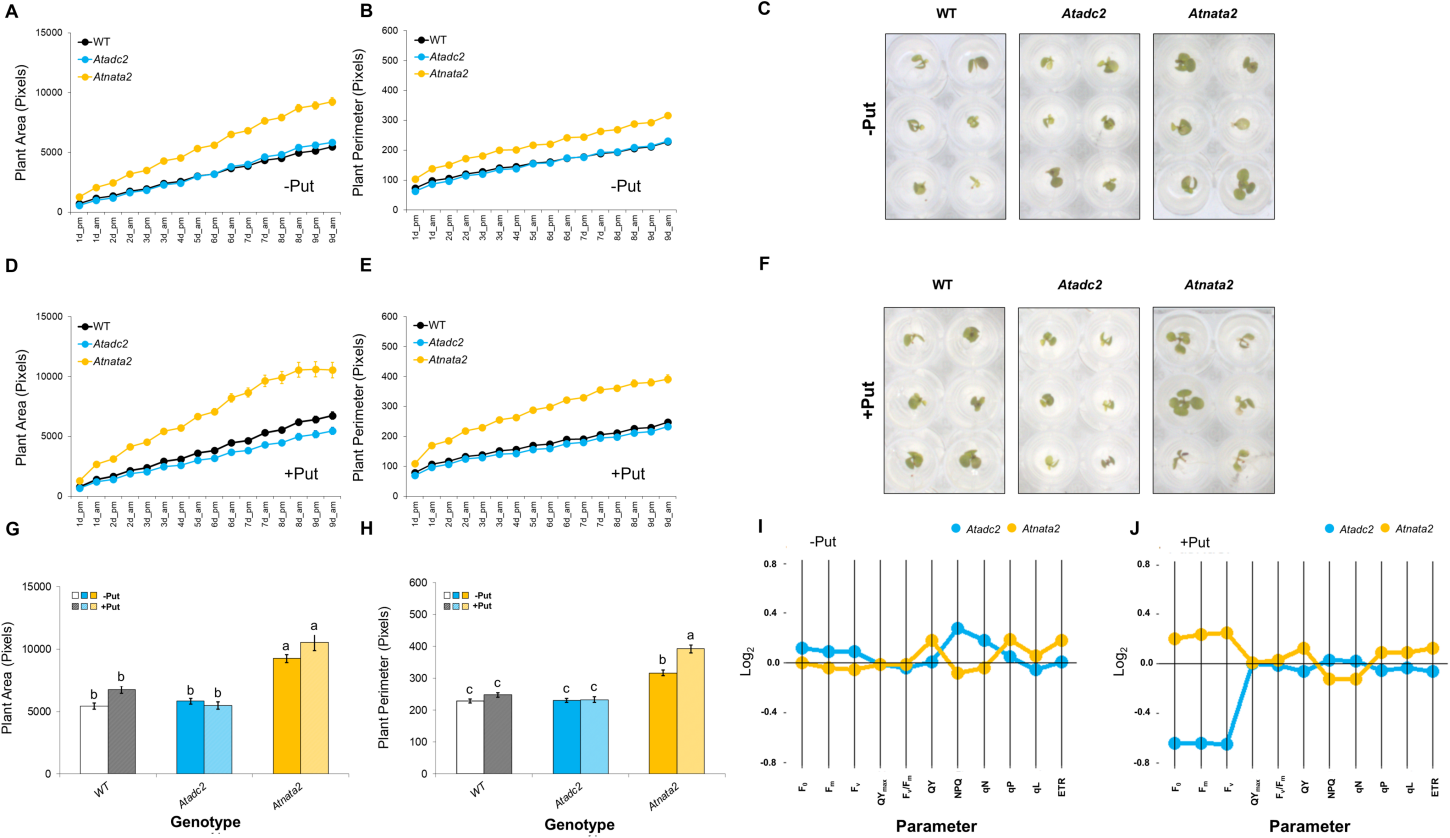
**Effect of Put priming on the biomass production and chlorophyll fluorescence‐related parameters in WT and the *Atadc2* and *Atnata2* mutant lines under long‐term saline stress.** Phenotyping analysis of Arabidopsis mutant lines *Atadc1*, *Atadc2*, *Atnata1*, and *Atnata2* and WT under long‐term saline stress (100 mM NaCl) without and with Put‐priming (0.1 mM Put) conditions. Pictures were taken during 7 days after 3 days of priming. The plant area (pixels), plant perimeter and the representative RGB images of the last day of the experiment of rosettes from **(A‐C)** non‐primed and **(D‐F)** Put‐primed seeds are represented. **(G‐H)** The plant area (pixels) and plant perimeter (pixels) of the last day measurement. Chlorophyll fluorescence parameters from **(I)** non‐primed and **(J)** Put‐primed seeds were analyzed on the last day of the experiment. The values represent the average of the 96 biological replicates per treatment. Statistical analysis was performed using two‐way ANOVA (*p* < 0.05) followed by a Tukey's test.

Clearly, the *Atadc* and *Atnata* mutant lines exhibited distinct metabolism alterations, influencing their growth, development, and response to Put priming. To explore these differences further, we selected the *Atacd2* and *Atnata2* lines, which displayed opposite behaviors, and grew them under salt stress with and without previous seeds priming with Put. The phenotype of the mutant lines under salinity mirrored the trends observed in the seedlings grown under optimal conditions with or without Put priming (Figure 4). The *Atadc2* mutant line did not show significant differences in rosette area and perimeter compared to WT under salt stress (Figure 4A‐C). However, the *Atnata2* line showed the best growing curve (represented as AUC, Supplementary Table S3), ending with a 93% larger rosette area and 68% bigger perimeter than WT under salinity (Figure 4A‐C and G). Seed priming with Put slightly alleviated the salt‐negative effect on the rosette growth (area and perimeter) in WT without significant differences but did not change it in the *Atadc2* mutant line (Figure 4D‐F and H). Only the *Atnata2* plants responded positively to Put‐priming, increasing the biomass even more (Figure 4D‐F and H, Supplementary Table S3B).

Salt stress also inhibited seed germination in all lines, particularly in *Atadc2* (Figure S2). Put priming significantly enhanced the seed viability (%) in the *Atadc2* and *Atnata2* mutant lines but did not affect WT. However, the improvement in seed germination in *Atadc2* did not translate into better seedling growth, reinforcing the idea that Put‐enhanced *At*ADC1 upregulation is more critical for seed germination than seedling growth in Arabidopsis.

### 3.8 Put priming enhanced light‐harvest by PSII in the *Atnata2* mutant line under salt stress

Salt stress affected fluorescence‐related parameters in all lines except for QY_max_ (Supplementary File S1B). The *Atnata2* mutant lines exhibited the highest F0, Fm, Fv, QY, qL, and ETR values and the lowest NPQ and *q*_N_ under salinity (Figure 4H). In contrast, the *Atadc2* mutant line showed the highest energy non‐photochemical dissipation (NPQ and *q*_N_). Seed priming with Put did not significantly alter the fluorescence‐related parameters in any line compared to their untreated counterparts (Supplementary File S1B). However, Put‐primed *Atadc2* seedlings reduced specific parameters such as F_0_, F_m_, F_v_, and QY (Figure 4I, Supplementary File S1B). Overall, the *Atnata2* mutant line performed better under salt stress due to a more efficient light capture, which could contribute to better growth under these conditions. Conversely, Put priming negatively impacted the *Atadc2* mutant line under salt stress, suggesting that the priming was detrimental rather than beneficial for this line in these growth conditions.

### 3.9 Put priming altered oppositely the N‐containing metabolites in the *Atadc2* and *Atnata2* mutant lines under salt stress conditions

**FIGURE 5:**
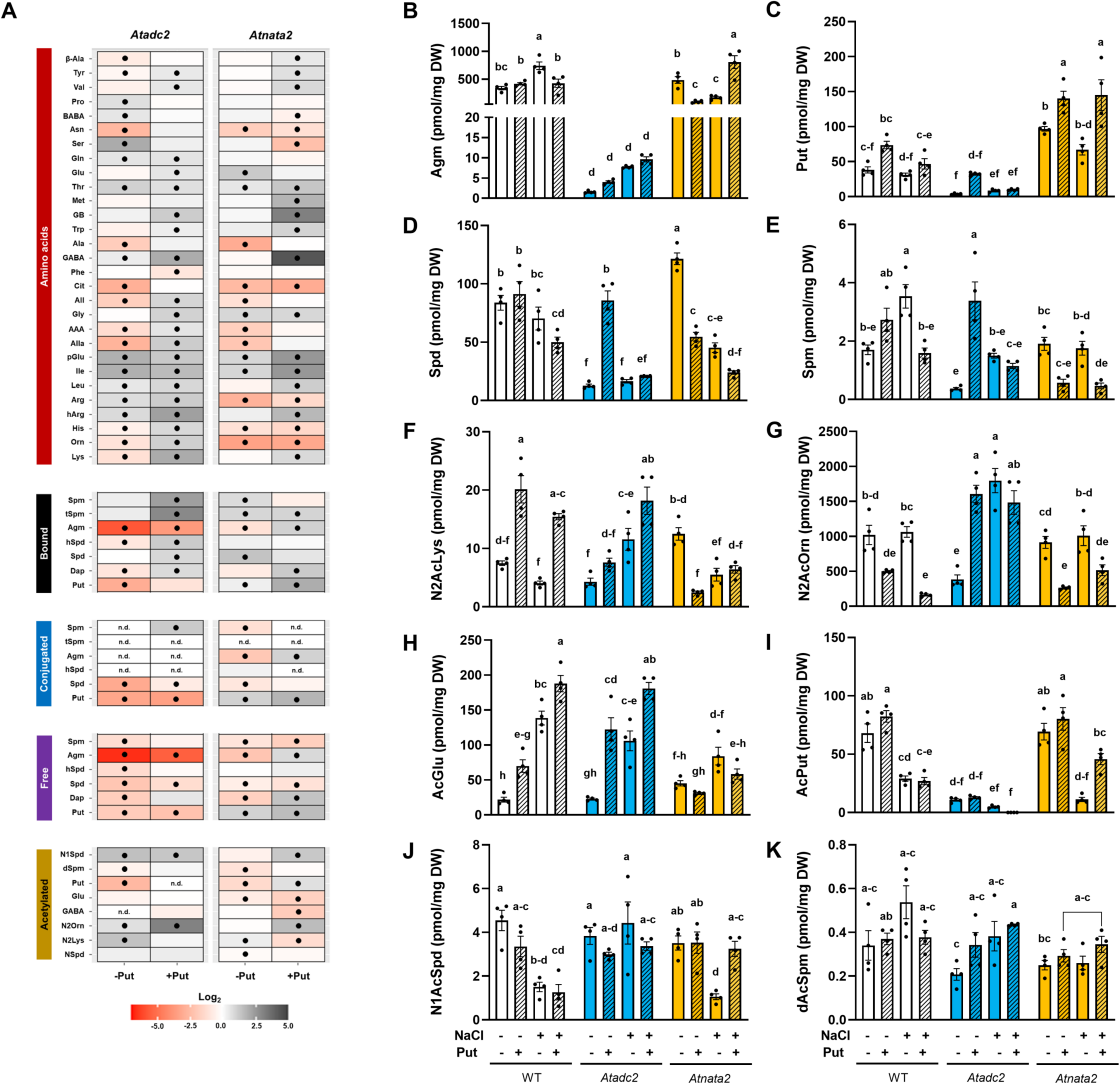
**Metabolic changes induced by Put priming in the WT, and the two mutant lines, *Atadc2* and *Atnata2*, under long‐term saline stress.** Metabolite content related to N‐containing metabolites was analyzed in the rosettes of three Arabidopsis lines: one WT (Col0) and two mutant lines (*Atadc2* and *Atnata2*) with or without priming with 0.1 mM Put under salt stress conditions. Heatmap (A) representing the changes of free amino acids [β‐alanine (β‐Ala), tyrosine (Tyr), valine (Val), proline (Pro), β‐aminobutyric acid (BABA), asparagine (Asn), serine (Ser), glutamine (Gln), glutamic acid (Glu), threonine (Thr), methionine (Met), betaine (Bet), tryptophan (Trp), alanine (Ala), γ‐aminobutyric acid (GABA), phenylalanine (Phe), citrulline (Cit), glycine (Gly), aspartic acid (AAA), D‐aspartic acid (DAA), pyroglutamic acid (pGlu), isoleucine (Ile), leucine (Leu), arginine (Arg), homo‐arginine (hArg), histidine (His), ornithine (Orn), lysine (Lys)] and bound, conjugated, free and acetylated polyamines [putrescine (Put), spermidine (Spd), spermine (Spm), 1,3‐diaminopropane (DAP), homospermidine (hSpd)] in the *Atadc2* **(A)** or *Atnata2* **(B)** mutant lines compared to WT without (−) or with (+) Put priming under control (first columm) or salt stress conditions (Second columm). The asterisk (*p* ≤ 0.05*) indicates significant differences between the mutant and WT lines. Changes in the content of Agm **(C)**, Put **(D)**, Spd **(E)** Spm **(F)** N2AcLys **(G)**, N2AcOrn **(H)**, AcGlu **(I)**, AcPut **(J)**, N1AcSpd **(K)** and dAcSpm **(L)** levels in WT and the *Atadc2* and *Atnata2* mutant lines with or without Put priming under control or salt stress conditions (100 mM NaCl). Different letters indicate significant differences according to Tukey's test after two‐way ANOVA (*p* < 0.05). Mean ± SE; n = 4 (each biological replicate consisted of an independent pool of 24 seedlings).

Interestingly, salt stress reduced several free amino acids, including Asn, Ala, All, 2‐aminoadipic acid (AAA), Alla, Cit, His, and Orn in the *Atadc2* and *Atnata2* mutant lines compared to WT (Figure 5A, Supplementary File S2B). The *Atadc2* seedlings also accumulated significantly higher levels of Gln than WT, resulting in a Gln/Glu ratio approximately double that of WT in these growth conditions (around 44 and 24, respectively). The *Atadc2* mutant line had lower Lys under salinity, whereas the *Atnata2* line reduced Arg under salinity, indicating a decrease in polyamine biosynthesis, reducing free and conjugated forms and the bound ones in *Atadc2*. In contrast, the *Atnata2* mutant line increased the bound tSpm, Spm, Put, and Spd, the most abundant Arabidopsis forms (Figure 5A, Supplementary File S2B).

Seed priming with Put altered this scenario significantly, enhancing the content of many free amino acids in both lines (Figures 5B, Supplementary File S2B). Focusing on amino acids related to polyamines, seed priming with Put enhanced the Arg, Orn, Lys, and GABA in the *Atadc2* mutant line compared to the WT under salt stress (Figure 5B and F, Supplementary File S2B). However, both lines reduced the GABA content when plants were grown with combined Put‐priming and salinity, highly increasing the Glu/GABA ratio (Supplementary File S2B). Contrarily, the *Atnata2* mutant line increased the GABA content under these conditions, keeping similar levels of Glu/GABA (Supplementary File S2B). In the *Atnata2* mutant line, Cit, Arg, and Orn levels remained lower than WT, resulting in reduced content of polyamines such as free tSpm, Spm, and hSpd (Figure 5B). However, this line enhanced the free Agm and Put content as a priming response under salt stress conditions (Figure 5B‐D). As expected, the *Atadc2* line maintained a low content of free Agm and Put under any circumstance and free Spd and Spm under salt stress, regardless of the priming (Figure 5A‐F). These results confirm that seed priming with Put induces opposite responses among mutant lines, particularly affecting nitrogen and its connection with polyamine metabolism, which could influence tolerance and growth capacity under salt stress conditions.

### 3.10 Put priming and salt stress altered the content of acetylated compounds differently in the lines

Salt stress also altered the acetylate compound content differently across the lines (Figure 5). For instance, WT increased AcGlu levels and reduced AcPut and N1AcSpd under salt stress. This trend persisted in the primed seeding, which exhibited even higher AcGlu levels (Figure 5H‐J). Additionally, Put priming induced N2AcLys accumulation in WT under salt stress to levels similar to those observed under control conditions (Figure 5F), indicating that this increase is likely a response to Put priming. Contrarily, regardless of growth conditions, N2AcOrn content decreased in WT and the *Atnata2* mutant as a priming response. However, the *Atadc2* line accumulated this acetylated form and AcGlu due to both priming and growth conditions (Figure 5A, G, Supplementary File S2B). The non‐primed *Atnata2* mutant line also reduced N2AcLys, AcPut, and N1AcSpd under salt stress, but Put priming enhanced the AcPut and N1AcSpd content to the basal levels (Figure 5G‐J). It is worth mentioning that the *Atadc2* mutant line consistently exhibited low AcPut content under all tested conditions and no significant changes in N1AcSpd and dAcSpm (Figure 5I‐K, Supplementary File S2B). Altogether, it is evident that some acetylated forms changed due to the priming effect while others were stress responses, with the most variable changes observed in the *Atadc2* mutant line.

### 3.11 The *Atadc2* and *Atnata2* mutant lines showed different expression patterns of many polyamine‐related genes after Put priming and under salt stress

**FIGURE 6:**
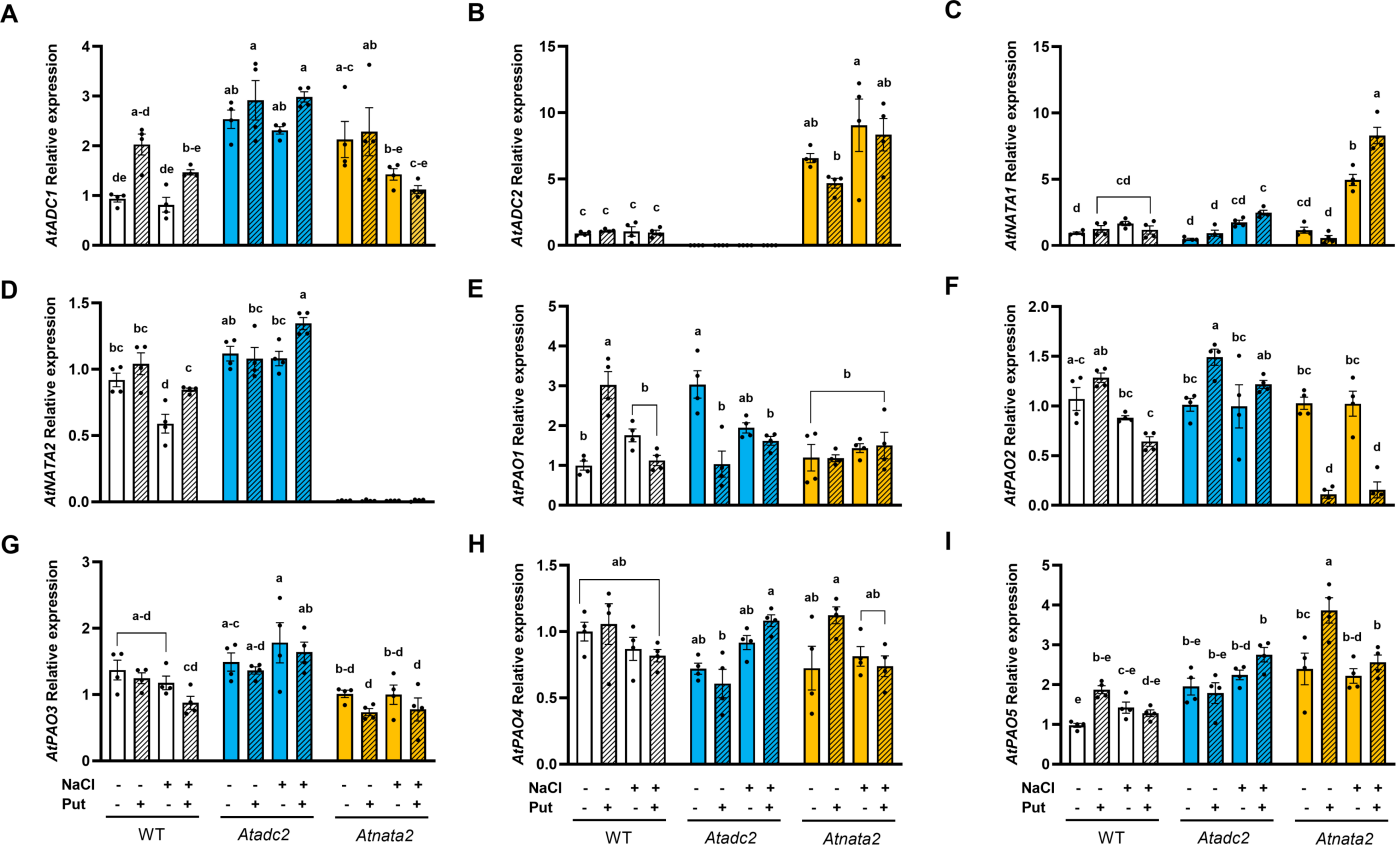
**Changes in the gene expression level of *AtADC*, *AtNATA,* and *AtPAO* genes in WT and the *Atadc2* and *Atnata2* mutant lines after Put priming under long‐term saline stress.** Relative gene expression of *AtADC1* **(A)**, *AtADC2* **(B)**, *AtNATA1* **(C)**, *AtNATA2* **(D)** and the five *AtPAO* family members (*AtPAO1‐5*, **E‐I**) analyzed by qPCR in WT and two Arabidopsis mutant lines (*Atadc2* and *Atnata2*) without (−) or with (+) 0.1 mM Put priming under control or salt stress (100 mM NaCl) conditions. *AtUBQ10* gene was used as a reference, and gene expression values were calculated using the 2^−∆∆Ct^ method. Different letters indicate significant differences according to Tukey's test after two‐way ANOVA (*p* < 0.05). Mean ± SE; n = 4 (each biological replicate consisted of an independent pool of 24 seedlings).

To understand further the metabolic changes in the lines, we analyzed the expression of several genes, including *AtADC1* and *AtADC2, AtNATA1* and *AtNATA2*, and the five *AtPAO* family members (*AtPAO1‐5*) (Figure 6, Supplementary File S3). In WT plants, notable changes were observed in *AtADC1*, *AtNATA2, AtPAO1,* and *AtPAO2* (Figure 6A, D‐F, Supplementary File S3). While *AtADC1* and *AtNATA2* expression tended to increase in response to the priming, *AtPAO2* expression was mainly reduced due to the salt stress in WT plants. Interestingly, *AtPAO1* gene expression was only enhanced in Put‐primed WT seedlings without stress.

The mutant lines exhibited different gene expression patterns. In the *Atadc2* mutant line, only the *AtPAO1* and *AtPAO2* gene expression showed significant change, with *AtPAO1* decreasing and *AtPAO2* increasing in response to the Put priming (Figure 6E, F, Supplementary File S3). The most notable finding was the overexpression of *AtADC2* in the *Atnata2* mutant line, which was five to ten times higher than in WT under all conditions (Figure 6B). The highest *AtADC2* upregulation in the *Atnata2* line happened under salinity while downregulating *AtADC1* (Figure 6A, B, Supplementary File S3). This line also increased *AtNATA1* gene expression as a salt stress response and reduced *AtPAO2* in the Put‐primed seedlings, independent of growth conditions (Figure 6C, F). Additionally, the *AtPAO5* expression pattern was opposite to the *AtADC2* gene expression in the *Atnata2* mutant line (Figure 6B, I).

### 3.12 Multivariate statistical analysis revealed a fine‐tuning between polyamine synthesis, acetylation, and catabolism regulating plant light harvest and growth

**FIGURE 7:**
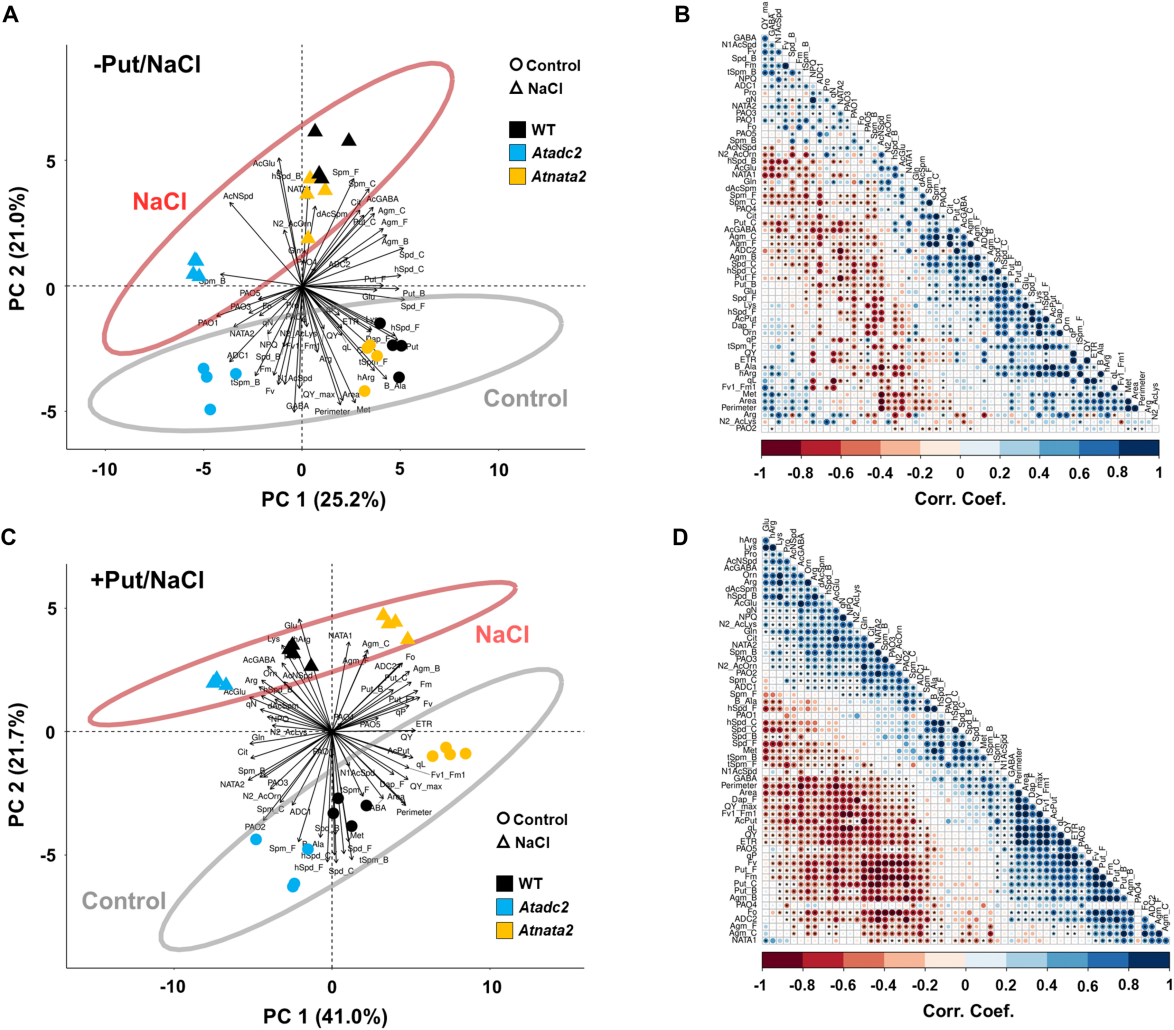
**Multivariate statistical analysis of metabolites, phenotyping, and chlorophyll fluorescence parameters in the WT and the two mutant lines, *Atadc2* and *Atnata2,* primed with Put under long‐term saline stress.** Principal component (PC) analyses **(A‐C)** and correlation matrices **(B‐D)** of the N‐containing metabolites, rosette area and perimeter, and chlorophyll fluorescence‐related parameters in WT and two Arabidopsis mutant lines (*Atadc2* and *Atnata2*) without **(A‐B)** or with **(C‐D)** 0.1 mM Put priming under control or salt stress conditions. The percentages of total variance represented by PC1 and PC2 are shown in parentheses.

Additional PC analyses and correlation matrices were performed to understand further the impact of Put priming on Arabidopsis growth under stress conditions. Firstly, the salt stress response of the three selected lines was analyzed by combining phenomic and metabolomic data with the gene expression without Put priming (Figure 7A, B). The two PCs, which explained 46.2% of the total model variation (PC1 = 25.2%; PC2 = 21%), mainly separated the control and salt‐stressed plants. WT and the *Atnata2* mutant line showed the highest dimensional separation and opposite response when grown under control or salt stress conditions, whereas the *Atadc2* line had less dimensional separation between the plants under control and salt stress conditions (Figure 7A). WT and the *Atnata2* exhibited the highest biomass, represented by larger rosette area and perimeters. These traits positively correlated with specific fluorescence‐related parameters such as QY_max_, F_v_’/F_m_′, ETR, and QY, and the accumulation of Met, hArg, βAla, or AcPut and reduction of AcGlu, N2AcOrn, AcNSpd. The *Atadc2* mutant line showed higher *AtNATA2* and *AtADC1* gene expression under control conditions, which correlated with the upregulation of *AtPAO1* and *AtPAO3*, and reduction of the *AtADC2* gene expression and content of many free polyamines including Agm, Put, Spd, and Spm, pointing to *AtNATA2* and *AtADC1* as possible regulators of polyamine catabolism (Figure 7A, B). Additionally, the *AtNATA2* gene expression was negatively related to fluorescence parameters associated with PSII light harvest (reduced QY, ETR, *q*_L_) and positively with the non‐photochemical parameters (NPQ and *q*_N_) (Figure 7A, B). These findings indicate that a fine‐tuning between polyamine synthesis, acetylation, and catabolism regulates light harvest efficiency in Arabidopsis.

Put priming significantly modified the response to salt stress, especially in the *Atadc2* and *Atnata2* mutant lines, as shown by PC1 and PC2, which captured 62.7% of the experimental model variation (41% and 21.7%, respectively) (Figure 7C). In this case, the *Atadc2* mutant line was closer to WT, while the *Atnata2* seedlings were more separated. Under salinity, the *Atadc2* mutant line and WT had the lowest biomass production (smaller rosette area and perimeter) due to a lower light harvest efficiency, indicated by lower photochemical and higher non‐photochemical parameters (Figure 7C). Contrarily, the *Atnata2* mutant line performed better under salt stress due to more efficient photosynthesis, as defined by the fluorescence‐related parameters such as F_0_, F_v_, F_m_, *q*_P_, ETR, QY, or *q*_L_. This line also showed higher gene expression of *AtNATA1* and *AtADC2* and accumulated all forms of Agm and Put, with these parameters significantly correlated (Figure 7C, D). Interestingly, metabolites like free tSpm and GABA were accumulated mainly in the non‐stressed *Atnata2* mutant line, which had the largest size and the best photosynthesis performance (Figure 7C, D).

## 4 DISCUSSION

Polyamines are promising SMBBs with significant potential in agriculture to enhance plant growth and resilience. Put has been extensively studied for its role as a crucial molecule in polyamine and nitrogen metabolism pathways and its positive effects against abiotic stress. However, most studies have used pre‐treatments and have exposed the plants to Put for prolonged periods with limited biological replicates, leading to controversial results. Our previous studies using high‐throughput phenotyping tools for rapid scoring of various growth and development features, combined with other omic approaches, have highlighted Put as a very promising SMBB for use as a seed priming agent to enhance plant growth, yield, and quality under stress conditions (Ugena et al., 2018; Hernandiz et al., 2022a). Despite these findings, the precise mode of action activated by this SMBB in plants remains unclear.

Put is a precursor for synthesizing higher polyamines such as Spd, Spm, hSPd, and tSpm. Additionally, Put can be acetylated, catabolized, conjugated, and capable of binding some macromolecules. We studied the effects of Put priming on the *Atadc1* and *Atadc2* mutant lines, which are well‐characterized for their development and seed germination impairments, leading to reduced responses to salt stress (Hummel et al., 2004; Urano et al., 2004, 2005). The *Atadc2* mutant line is known to have low Put endogenous levels. Furthermore, Arabidopsis, like other brassicas, lacks ODC enzymes that contribute to polyamine synthesis. However, plants may have an additional pathway for polyamine synthesis via NATAs (Lou et al., 2020), although these enzymes' function and regulatory role in polyamine metabolism are still unknown. Understanding how Put and other polyamines influence plant stress responses could pave the way for more targeted and effective use of SMBBs in agriculture.

We included the *Atnata1* and *Atnata2* single mutants in our study to deepen our understanding. Recently, *At*NATA1 has been characterized and shown to acetylate a broad spectrum of compounds, including Put. Some reports suggested that *At*NATA1 regulates the bioavailability of free Put, preventing its catabolism by *At*PAOs and, hence, regulating reactive oxygen species (ROS) production (Lou et al., 2016). However, this connection between *At*NATAs and *At*PAOs has not been proven. This study characterized these four Arabidopsis mutant lines (*Atadc1, Atadc2, Atnata1,* and *Atnata2*) under seed priming with Put to understand their role in regulating plant growth enhancement under control and salt stress conditions. It provides a comprehensive biochemical characterization of the polyamine pathway, from synthesis to catabolism, to clarify the cross‐regulation between these processes.

### 4.1 The loss‐of‐function of *AtADC2* gene causes insensibility to Put priming

Seed priming with Put enhanced plant growth and fitness more effectively than other polyamines in *Arabidopsis thaliana* (Ugena et al., 2018; Hernándiz et al., 2022a) and other plant species (Doneva et al., 2021; Ghahremani et al., 2023; Jalili et al., 2023.; Hernandiz et al., 2022b). *AtADC2* is a crucial gene in seed development, particularly during the early stages of embryo, endosperm, and funiculus development. However, its role in regulating seedling growth has not been proved essential. Firstly, we corroborated that the *Atadc2* mutant line has reduced Put levels compared to WT as shown by Urano et al. (2004, 2005). This line did not respond to Put priming, so the seedling did not increase the rosette size (Figure 1). Conversely, the rosette size of the *Atnata2* mutant line was significantly larger than that of WT, particularly under Put priming (Figure 1). Interestingly, the *Atnata2* mutant line accumulated significantly higher Put levels and showed upregulated *AtADC2* gene expression under all growth conditions compared to WT (Figures 5C and 6B). These results and the morphological, physiological, and metabolic differences between the *Atadc2* and *Atnata2* mutant lines underscored a possible inverse crosstalk regulating plant growth, particularly under Put priming.

### 4.2 *At*ADC2 and *At*NATA1 are important in regulating nitrogen metabolism, and the biosynthesis, catabolism and acetylation of polyamines under Put‐priming

Put priming significantly altered the metabolic profile of the plants, with different effects observed in the mutant lines. Put is central to various critical pathways, including terminal catabolism leading to GABA production and serving as a substrate for *At*NATA1 to produce AcPut (Lou et al., 2016, 2020). In this context, the low Put production in the *Atadc2* mutant line also predicted reduced GABA levels. However, these seedlings showed a higher GABA and lower Glu content than WT under all growth conditions (Supplementary File S2). The *Atadc2* mutant line also exhibited the highest Gln and Arg accumulation under Put priming and salt stress conditions. This may suggest a possible imbalance in plant nitrogen status since this shift toward high N: C ratios is typically observed under altered N conditions (Ruan et al., 2010; Gent & Forde, 2017). Arg, in particular, has the highest N:C (4:6) ratio among all amino acids and is the primary nitrogen storage and transport molecule [reviewed by Siddappa & Marathe (2020)]. Arg catabolism, linked to urea production, is also crucial for avoiding ammonium toxicity in plants (Siddappa & Marathe, 2020; Urra et al., 2022).

Similarly, converting Glu to Gln is also vital for maintaining ammonium homeostasis; its disruption leads to rapid accumulation and plant death (Bittsánszky et al., 2015). In our work, the elevated Gln levels in the *Atadc2* mutant line resulted in a very high Gln/Glu, suggesting that this line may have upregulated the glutamine synthetase enzymes (*At*GLN, E.C. 6.3.1.2), which convert Glu to Gln, particularly *At*GLN2 in the chloroplast, to prevent ammonium accumulation. Since ammonium accumulation is more pronounced in salt‐stress plants, particularly in susceptible cultivars (Zhou et al., 2004), the increase in Arg and Gln in the *Atadc2* mutant line under salinity likely indicated a higher stress level. Furthermore, a recent study reported that excessive ammonium assimilation by *At*GLN2 also produces significant proton levels, aggravating the acidic burden and leading to plant toxicity (Hachiya et al., 2021), which could have imposed even deeper stress on *Atadc2* seedlings.

Interestingly, the Put priming did not reduce the Gln/Glu ratio observed in this line but enhanced the Spd and Spm levels under control conditions. The *Atadc2* mutant line also exhibited deficient levels of Agm regardless of the Put‐priming (Figure 2B‐C), indicating that *At*ADC2 is the main enzyme that produces Put via Arg decarboxylation. This deficiency compromises the synthesis of the superior polyamines (Figure 2D‐E). Additionally, almost no changes were observed in the *AtPAO* gene expression, suggesting that the increased levels of Spd and Spm in Put‐primed *Atadc2* seedlings likely result from *de novo* synthesis from the exogenously added Put. Further studies on the crosstalk between *AtADC* genes and nitrogen and polyamine metabolism are needed to understand these complex interactions and their role in controlling ammonium toxicity and plant growth.

Without priming, the *Atadc2* mutant line also showed reduced levels of AcOrn and AcPut compared to WT under control conditions (Figure 2H, J). However, Put‐primed *Atadc2* seedlings enhanced the content of Spd and Spm, AcOrn, and AcGlu, but not AcPut. *At*ADC1 enzyme converts AcOrn into AcPut (Lou et al., 2020), explaining the coordinated changes in the *AtADC1* gene expression and AcOrn content in the WT (Figures 2, 5, and 6). The *Atadc2* mutant line consistently showed low AcPut levels in all tested variants (Figure 5I), even with higher *AtADC1* gene expression levels (Figure 6A). These findings suggest that *At*ADC1 requires *At*ADC2 to convert AcOrn into AcPut, possibly through enzyme activity regulation. It is worth mentioning that the *At*ADC1 and *At*ADC2 enzymes are located in different cellular compartments (Maruri‐López & Jiménez‐Bremont, 2017; Lou et al., 2020). However, they can interact to form homodimers or heterodimers in the cytoplasm and chloroplast (Maruri‐López & Jiménez‐Bremont, 2017). This complex formation may be essential for proper enzymatic activity, highlighting the importance of their interaction for effective polyamine synthesis and regulation.

Regarding AcOrn, it is synthesized from Orn acetylated by the *At*NATA1 action (Lou et al., 2020). Significant correlations between *AtNATA1* and *AtADC2* gene expression and *AtNATA2* and *AtADC1* suggest coordinated regulation between these enzyme pairs. *At*ADC1 and *At*NATA1 likely work together in the endoplasmic reticulum (ER) (Lou et al., 2020), while *At*ADC2 and *At*NATA2 are predicted to be located in the chloroplast (Figure S4). These enzyme pairs may regulate each other to determine the pathway contributing to polyamine synthesis and plant growth. For instance, the increase in AcGlu observed, especially in the *Atadc2* mutant line as a priming effect and salt stress response, contributes to Orn synthesis via Glu, which changed in parallel with AcOrn (Figure 5I, G). However, accumulating these metabolites did not translate into better growth in this mutant line. This may be due to the inability to synthesize AcPut via *At*ADC1, as mentioned above. It is important to consider that while the accumulation of certain intermediates such as AcGlu and AcOrn indicates active metabolic pathways, the functional outcome, such as enhanced growth, depends on the efficient downstream conversion to necessary metabolites like AcPut. The deficiency of the *Atadc2* mutant line in converting these intermediates to concrete metabolites due to impaired *At*ADC2 activity suggests that a coordinated interaction between various enzymes in the polyamine biosynthesis pathway is crucial for achieving optimal growth and stress tolerance. Moreover, these results highlight the necessity of a functional *AtADC2* gene for proper polyamine homeostasis, impacting nitrogen assimilation and plant ability to cope with stress.

A strong positive correlation was also observed between the *AtADC1* and *AtNATA2* gene expression levels and certain *AtPAOs* (Figure 7B, D). For instance, the *Atadc2* mutant line had reduced free Put, Spd, and Spm levels, with Dap and tSpm under the limit of detection (Figure 2), and upregulated the *AtPAO1* gene expression levels (Figure 6E). Put‐priming enhanced the polyamine levels to the WT under the same conditions, except Agm, and reduced *AtPAO1* gene expression levels, indicating that this enzyme may be the primary catabolic regulator of free polyamines like Spd, Spm or tSpm. Conversely, the *Atnata2* mutant line, which showed the largest rosette size and further increased after Put‐priming, had the highest *AtADC2* gene expression levels, which also correlated to *AtNATA1* gene expression and many forms of Agm and Put. Furthermore, after Put‐priming, this line accumulated even higher Put levels and reduced the content of Spd and Spm, probably due to the *At*PAO4 and *At*PAO5 activity (Figures 5C‐E and 6H and I). These results demonstrate that the *AtNATA* genes are not redundant, and *AtNATA2* likely plays a more significant regulatory role in plant growth than *At*NATA1 enzyme. In this regard, it has been proved that recombinant *At*NATA2 prefers to acetylate Dap and thialysine (Lou et al., 2016). However, the positive correlation between the *AtNATA2* gene expression and the content of AcGlu, AcGABA, and some acetylated forms of Spd and hSpd suggest other possible free substrates for this enzyme. Further characterization is needed to corroborate these findings.

### 4.3 The loss‐of function of *AtNATA2* gene led to Put production by *At*ADC2, which improves light harvest, enhancing growth and salt tolerance

It is well known that the exogenous application of polyamines can alleviate the negative effect of abiotic and biotic stress, and specifically, Put has been shown to enhance salt tolerance in various plant species (Ghahremani et al., 2023; Jalili et al., 2023; Hernándiz et al., 2022a; Sequera‐Mutiozabal et al., 2017; Zhao et al., 2023). Our studies also showed that Put priming enhanced plant growth in all plants except for the *Atadc2* mutant line, indicating Put insensibility. Moreover, priming seeds with Put enhanced greenness under normal and salt stress conditions in Arabidopsis (Ugena et al., 2018; Hernándiz et al., 2022a). One reason is that the treatment with Put improved the light harvest (higher QY and lower heat dissipation), particularly in the *Atnata2* line, but did not help the *Atadc2* mutant line under salt stress (Figure 4J). Furthermore, *AtADC2* is significantly upregulated in the *Atnata2* mutant line compared to WT and accumulated considerably higher levels of Put with priming (Figures 2D and 6B). Shu et al. (2015) demonstrated that exogenous Put application alters unsaturated fatty acid content in plants under salt stress, thereby alleviating the disintegration of thylakoid grana lamellae and reducing the number of plastoglobuli in thylakoid membranes. Furthermore, Put can regulate protein expression at transcriptional and translational levels by increasing endogenous polyamine levels in thylakoid membranes, which may stabilize photosynthetic apparatus under salt stress (Shu et al., 2015). This could explain the high bound and conjugated Put observed in the *Atnata2* mutant line (Figure 5A). Additionally, the Put accumulation in the thylakoid lumen during light reactions may act as a permeable buffer and an osmolyte, as was reported in tobacco leaf discs (Kotakis et al., 2014). Recent studies have shown that photosynthetic parameters such as F_v_/F_m_ increased along with pigment content in wheat seedlings while maintaining ion homeostasis under Put treatment and salt stress, resulting in enhanced plant height and biomass (Zhao et al., 2023). This underscores the critical role of the *AtADC2* gene in facilitating the beneficial effect of Put on photosynthesis and stress tolerance.

## 5 CONCLUSION

Our research confirms that seed priming with Put is an effective small molecule‐based biostimulant (SMBBs), significantly enhancing plant growth and mitigating the adverse effects of salt stress. The insensitivity of the *Atadc2* mutant to Put priming underscores the crucial role of *At*ADC2‐mediated Put synthesis in the chloroplast for better growth and improving salt stress tolerance. Additionally, the highly upregulated expression levels of the *AtADC2* gene in the *Atnata2* mutant, regardless of the growth conditions and priming, suggests that *At*NATA2 acts as a negative regulator of *At*ADC2 and, consequently, Put synthesis in Arabidopsis. Furthermore, reduced Put levels in the chloroplast may impact light harvest in PSII, indicating an indirect control of *At*NATA2 of all this process that conditions plant growth.

These results indicate that plant growth and stress responses are not solely influenced by changes in free polyamines as substrates for enzyme metabolism but are regulated by a complex interplay of transcriptional and post‐transcriptional mechanisms. This regulatory crosstalk involves synthesis, acetylation, and catabolism processes that intricately interact. Understanding the regulatory mechanisms involving the crosstalk between *AtADC* and *AtNATA* genes, including the possible negative regulation of *At*ADC2 by *At*NATA2, in polyamine metabolism can lead to more targeted and effective agricultural practices. Based on our findings, seed priming with Put could create a somatic stress memory that enhances resilience to salt stress, offering a promising approach for improving crop performance under challenging environmental conditions. Increasing evidence highlights the positive effects of exogenous Put application on yield, abiotic stress tolerance, and post‐harvest longevity in various agriculturally significant plant species (Hernandiz et al., 2022b; Khosroshahi et al., 2007; Rahman et al., 2024). Current applications involve drenching, supplementation, and pre‐ and post‐treatment via foliar application. However, exploring the long‐term effects of seed priming with Put across different crops could be particularly beneficial, potentially reducing costs due to easier application and lower concentration requirements. This technology could pave the way for significant advancements in crop science and agricultural sustainability by elucidating the complex interactions between polyamine metabolism, nitrogen assimilation, and plant stress responses.

## AUTHOR CONTRIBUTIONS

FIJ‐R and NDD funded the project. FIJ‐R, CEA‐P, and NDD designed the experiments. FIJ‐R, CEA‐P, and IS‐F performed the experiments. FIJ‐R, CEA‐P, PK, IS‐F, and SĆZ performed phenotyping, molecular and biochemical determinations. FIJ‐R, CEA‐P, PK, and NDD performed the data analysis. All authors discussed the results. FIJ‐R, CEA‐P, SĆZ, and NDD wrote the manuscript. All authors agreed with the final version.

## FUNDING INFORMATION

This work was supported by the project JG_2024_036 implemented within the Palacký University Young Researcher Grant.

## Supporting information


**Appendix S1:** Supporting Information

**Supplementary File S1A:** Fluorescence‐related parameters determined on wild‐type (Col‐0), and four mutant lines (*Atadc1*, *Atadc2*, *Atnata1*, and *Atnata2*) with (+) or without (−) seed priming with 0.1 mM Put under control conditions. The study included 96 biological replicates.
**Supplementary File S1B:** Fluorescence‐related parameters determined on wild‐type (Col‐0), and two mutant lines (*Atadc2*, and *Atnata2*) with (+) or without (−) seed priming with 0.1 mM Put under salt stress conditions (100 mM NaCl). The study included 96 biological replicates.

**Supplementary File S2A:** Content of the acetylated polyamines and amino acids, free, conjugated, and bound polyamines and free amino acids content in wild‐type (Col‐0), and four mutant lines (*Atadc1*, *Atadc2*, *Atnata1*, and *Atnata2*) with (+) or without (−) seed priming with 0.1 mM Put under control conditions (full MS). The study included four biological replicates, constituted by independent pools of 24 seedlings each collected at the end of the experiment.
**Supplementary File S2B:** Content of the acetylated polyamines and amino acids, free, conjugated, and bound polyamines and free amino acids content in wild‐type (Col‐0), and two mutant lines (*Atadc2* and *Atnata2*) with (+) or without (−) seed priming with 0.1 mM Put under control conditions (full MS) or salt stress conditions (100 mM NaCl). The study included four biological replicates, constituted by independent pools of 24 seedlings each collected at the end of the experiment.

**Supplementary File S3:** The data shows the relative gene expression values of *AtADC*, *AtNATA*, and *AtPAO* genes in WT (Col‐0), and two mutant lines (*Atadc2 and Atnata2*) with (+) and without (−) seed priming with 0.1 mM Put under control (full MS) or salt stress (100 mM NaCl) conditions. The study included four biological replicates constituted by independent pools of 24 seedlings each.

## Data Availability

The data supporting this study's findings are available from the corresponding author upon reasonable request.
