# Supplementary material for: The loss‐of‐function of AtNATA2 enhances AtADC2‐dependent putrescine biosynthesis and priming, improving growth and salinity tolerance in Arabidopsis
Source: Physiol Plant. 2024 Nov 3;176(6):e14603. doi: 10.1111/ppl.14603 (PMC11659803; doi:10.1111/ppl.14603)
Supplement: Supplementary file 1 — Appendix S1: Supporting Information [file PPL-176-e14603-s002.pdf]

The loss-of-function of *AtNATA2* enhances *At*ADC2-dependent putrescine biosynthesis and priming, improving growth and salinity tolerance in *Arabidopsis thaliana*

Francisco Ignacio Jasso-Robles; Carlos Eduardo Aucique-Perez; Sanja Ćavar Zeljković; Iñigo Saiz-Fernández; Pavel Klimeš and Nuria De Diego.

Supplementary Table S1. The oligonucleotide sequences used to analyze the gene expression in WT and two Arabidopsis mutant lines (*Atadc2* and *Atnata2*).

Gene	AGI code	Oligonucleotide sequence		Amplicon (pb)	Reference
		Forward	Reverse		
AtUBQ10	AT4G05320	TAATCCCTGATGAATAAGTGTTCTAC	AAAACGAAGCGATGATAAAGAAG	98	Jasso-Robles et al. 2020
AtADC1	AT2G16500	GTGGTGATAAGGGGAACGACA	GTGGTGATAAGGGGAACGACA	199	Alcazar et al. 2006
AtADC2	AT4G34710	CGTGTGGTTTGTTGTTGCATTATATTAT	AAAGGCTTTCATAATTCGGATCTTCTT	148	This work
AtNATA1	AT2G39030	GAATGCTATCAACTTCTATGAGCAG	CGCTCGATGGGTCTCATGCA	142	This work
AtNATA2	AT2G39020	GGAAGAGTGGAATGGGTTGTTCT	CTCTCAGCAACACAGCATCAATC	168	This work
AtPAO1	AT5G13700	CTAAACCAGGTAGTTAGGGAG	GCTTCTGTTTTCCATCTGGGTAA	170	This work
AtPAO2	AT2G43020	GACCAGGAGGAATTGTTGCCT	CCTGACAATCTTAGTAACCCTGT	123	This work
AtPAO3	AT3G59050	ATAATCTATTCTTTGCGGGGGAAG	AAGCAGGAGCTTCCTCTTCCAT	159	Jasso-Robles et al. 2020
AtPAO4	AT1G65840	AAGGTATATCTTTGAACGACTCGG	GGAACCCTGCTTCTGTCTGTTA	148	Jasso-Robles et al. 2020
AtPAO5	AT4G29720	GAGGCTCCTATTCGTATGTAGC	CTTCCCCTGCAAACATGACTTG	153	Jasso-Robles et al. 2020

Supplementary Table S2. Area under the curve (AUC) calculated from the growing curve of the rosette area and perimeters (green pixels, Figure 1) in five different Arabidopsis lines (WT and four mutants) with or without seed priming with 100 μM putrescine (+Put or -Put, respectively) grown in full MS medium for eight days.

		AUC					
		Area			Perimeter		
**Line**	**Priming**	**Average**		**±SD**	**Average**		**±SD**
**WT**	**-Put**	88971	de	8.6	3449	de	54.1
**WT**	**+Put**	113527	c	6.8	4295	c	48.5
Atadc1	**-Put**	79456	e	6.6	3881	e	41.9
Atadc1	**+Put**	99215	cde	6.3	3789	cd	42.0
Atadc2	**-Put**	95501	cde	8.3	3789	cde	51.4
Atadc2	**+Put**	104215	cd	7.4	4123	c	50.3
Atnata1	**-Put**	108855	cd	8.0	4008	c	57.8
Atnata1	**+Put**	135434	b	9.9	4934	b	64.4
Atnata2	**-Put**	145178	b	8.8	5030	b	67.1
Atnata2	**+Put**	175732	a	8.0	6413	a	47.3

Supplementary Table S3. Area under the curve (AUC) calculated from the growing curve of the rosette area and perimeters (green pixels, Figure 3) in three different Arabidopsis lines (WT and two mutants, *Atadc2* and *Atnata2*) with or without seed priming with 100 μM putrescine (+Put or -Put, respectively) grown in full MS medium supplemented with 100 mM NaCl for inducing salt stress for eight days.

		AUC					
		Area			Perimeter		
**Line**	**Priming**	**Average**		**±SD**	**Average**		**±SD**
**WT**	**-Put**	46955	c	8.6	2356	c	54.1
**WT**	**+Put**	57465	c	6.8	2567	c	48.5
Atadc2	**-Put**	47998	c	6.6	2307	c	41.9
Atadc2	**+Put**	47442	c	6.3	2388	c	42.0
Atnata2	**-Put**	82537	b	8.3	3286	b	51.4
Atnata2	**+Put**	101776	a	7.4	4239	a	50.3

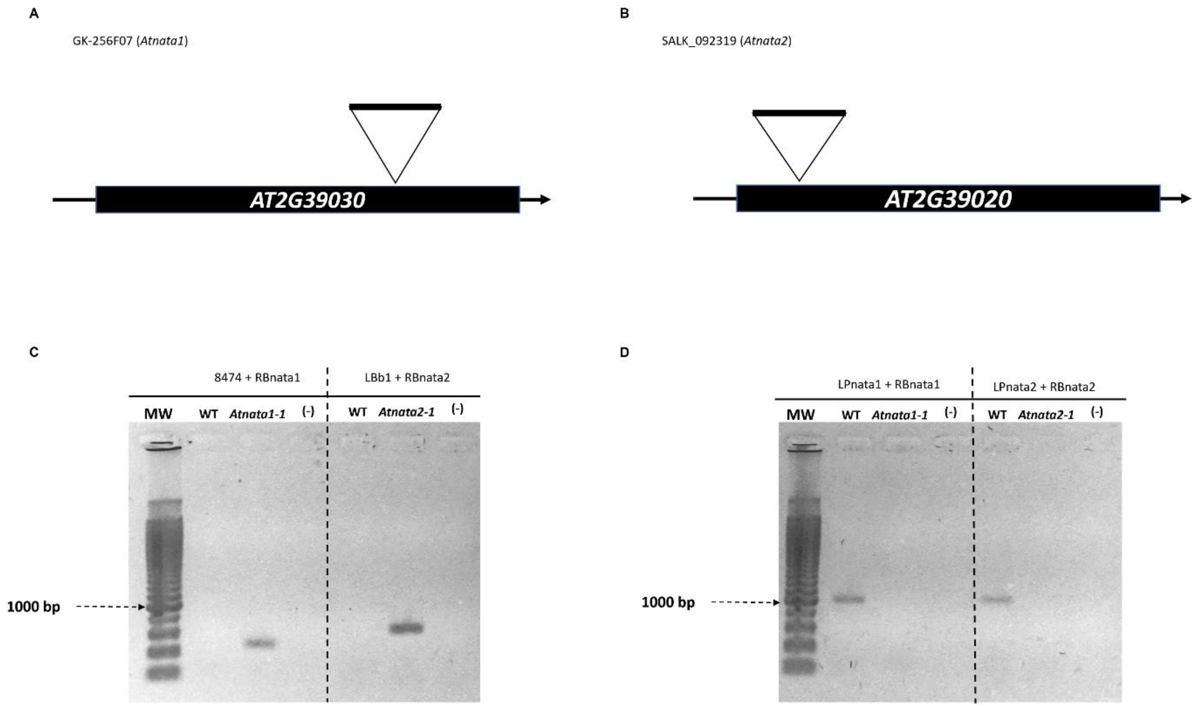

Supplementary Figure S1. Isolation and molecular characterization of *Atnata1-1* and *Atnata2-1* T-DNA insertion mutant lines. Structure of the *AtNATA1* (**A)** and *AtNATA2* (**B)** locus and the localization of the T-DNA insertion. Identification of the T-DNA insertion by PCR in WT, *Atnata1-1* (GK-256F07), and *Atnata2-1* (SALK_092319) mutant lines (**C**). The LbB1 T-DNA specific oligonucleotide and the RB gene specific oligonucleotide for each gene were used to identify the T-DNA insertion. To determine the homozygosis of the mutant lines, the RB and LP gene-specific oligonucleotides for each gene that are flanking the T-DNA insertion region were used (**D)**.

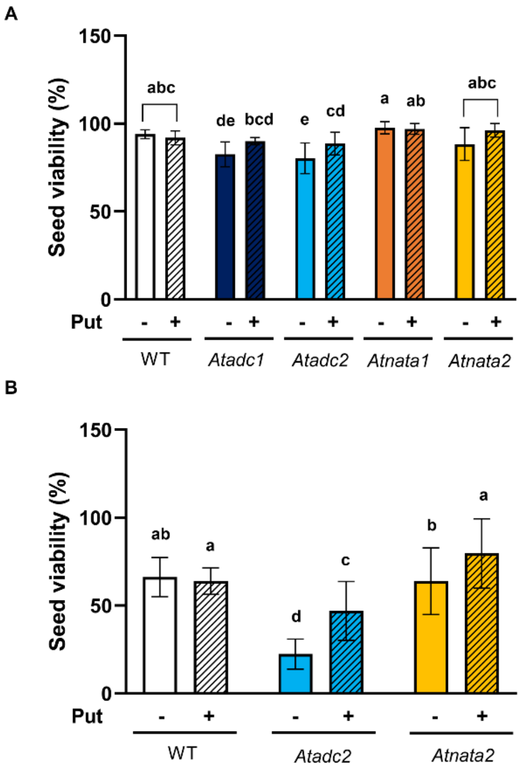

Supplementary Figure S2. Put priming affected the germination of WT and the *Atadc* and *Atnata* mutant lines. Seed germination of WT and four Arabidopsis mutant lines (*Atadc1*, *Atadc2*, *Atnata1*, and *Atnata2*) without or with 0.1 mM Put priming under control conditions **(A).** Seed germination of WT and two Arabidopsis mutant lines (*Atadc2*, and *Atnata2*) without or with 0.1 mM Put priming under control or salt stress conditions **(B).** Different letters indicate significant differences among variants according to Tuley's test after one-way ANOVA. Mean ± SE; n = 8 (each biological replicate consisted of an independent pool of 40 seeds).

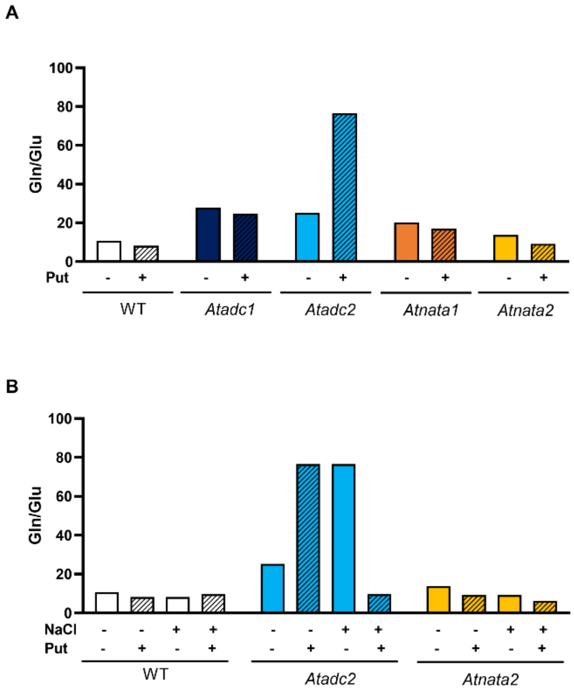

Supplementary Figure S3. Put priming differently altered glutamine/glutamate ratio in the *Atadc* and *Atnata* mutant lines. Gln/Glu ratio in WT and four Arabidopsis mutant lines (*Atadc1*, *Atadc2*, *Atnata1* and *Atnata2*) without (-) or with (+) Put priming under control conditions **(A).** Gln/Glu ratio in WT and two Arabidopsis mutant lines (*Atadc2*, and *Atnata2*) without (-) or with (+) Put priming under control or salt stress (100 mM NaCl) conditions **(B).**

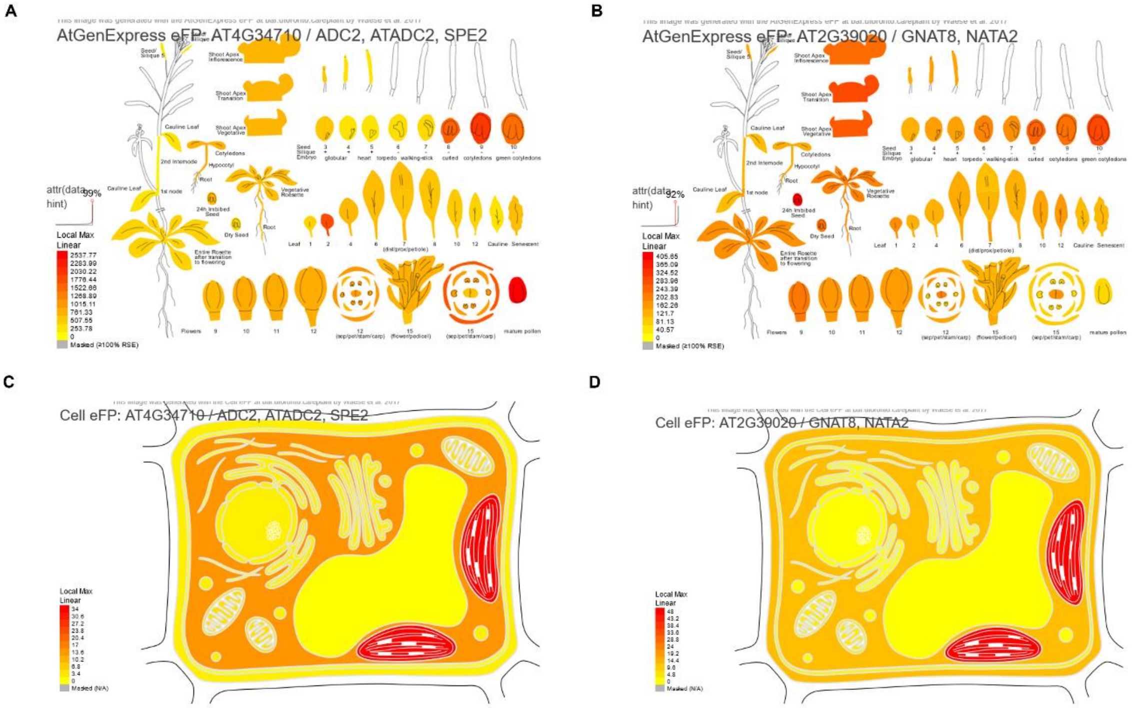

Supplementary Figure S4. eFP browser developmental and subcellular localization prediction view of *AtADC2* and *AtNATA2*. *AtADC2* **(A)** and *AtNATA2* **(B)** gene expression in different developmental stages. *At*ADC2 (**C)** and *At*NATA2 (**D)** subcellular localization. https://bar.utoronto.ca/eplant/
